# The West Pacific Gradient tracks ENSO and zonal Pacific sea surface temperature gradient during the last Millennium

**DOI:** 10.1038/s41598-021-99738-3

**Published:** 2021-10-14

**Authors:** J. Zinke, S. A. Browning, A. Hoell, I. D. Goodwin

**Affiliations:** 1 School of Geography, Geology and the Environment, University of Leicester, University Rd, Leicester, LE1 7RH UK; 2 Molecular and Life Sciences, Curtin University, Kent St, Bentley, Perth, WA 6102 Australia; 3 Australian Institute of Marine Science, Townville, PMB No.3, Townsville, QLD 4810 Australia; 4 School of Geography, Archaeology and Environmental Studies, University of Witwatersrand, Braamfontein, Johannesburg, 2000 South Africa; 5 Risk Frontiers, St. Leonards, NSW 2065 Australia; 6 Physical Sciences Laboratory, NOAA, Boulder, CO USA; 7 UWA Oceans Institute, University of Western, Crawley, WA 6009 Australia; 8 Climalab, Newport, NSW 2106 Australia; 9 Climate Change Research Centre, University of New South Wales, Sydney, NSW 2052 Australia

**Keywords:** Climate change, Palaeoceanography, Palaeoclimate

## Abstract

Small changes in Pacific temperature gradients connected with the El Niño Southern Oscillation (ENSO) influence the Walker Circulation and are related to global climate anomalies. Therefore, it is of paramount importance to develop robust indices of their past behavior. Here, we reconstruct the difference in sea surface temperature between the west and central Pacific during ENSO, coined the West Pacific Gradient (WPG), based on the Last Millennium Paleo Hydrodynamics Data Assimilation. We show that the WPG tracks ENSO variability and strongly co-varies with the zonal gradient in Pacific sea surface temperature. We demonstrate that the WPG strength is related to significant atmospheric circulation and precipitation anomalies during historical El Niño and La Niña events by magnifying or weakening droughts and pluvials across the Indo-Pacific. We show that an extreme negative WPG coupled to a strong zonal Pacific temperature gradient is associated with enhanced megadroughts in North America between 1400 CE and the late sixteenth century. The twentieth century stands out in showing the most extreme swings between positive and negative WPG conditions over the past Millennium. We conclude that the WPG is a robust index together with ENSO indices to reveal past changes in Pacific zonal sea surface temperature gradient variability.

## Introduction

A lack of long instrumental climate records from the Indo-Pacific warm pool and the tropical Pacific, the heat engines of the global climate system and an essential player in global rainfall/drought cycles, is a central problem for reducing uncertainties in model-based climate change process studies and projections to plan for the future successfully. The Maritime Continent (MC) plays a crucial part in the global hydrological variability through its influence on the Indo-Pacific Walker circulation^1^ collocating within the Indo-Pacific Warm Pool, where sea surface temperatures (SST) exceed 28 °C associated with intense deep convective rainfall year-round. The Maritime Continent includes Indonesia, Malaysia, New Guinea, and the surrounding shallow seas. The Maritime Continent is collocated within significant centers of high interannual SST variability in the Pacific on its margins. To the east, the western pole of the El Niño-Southern Oscillation (ENSO)-SST anomalies (Niño4 and Niño3.4 regions^2^) straddles the Maritime Continent and have the most substantial influence on Maritime Continent precipitation between austral winter and spring. The convective activity over the Maritime Continent associated the Madden Julian Oscillation and linkages with ENSO is associated to large-scale variations in the climate system and global rainfall-drought patterns in austral summer^1^. This includes the Asian Monsoon, African Monsoon, Austral-Asian Monsoon, and the American Monsoon systems, all intimately linked via changes in the Walker Circulation^3–6^. However, correctly simulating precipitation across the Maritime Continent in climate models and its impact on the Pacific Walker Circulation (hereafter PWC) presents a significant challenge due to its topographic complexity, often resulting in biased estimates of Maritime Continent convective activity^1,7^. The same holds for air temperatures across the Maritime Continent.

Conflicting evidence points to either a strengthening or weakening PWC over the twentieth century based on changes in SST and sea level pressure (SLP) gradients between the western and eastern Pacific^4,8–12^. The difficulty in establishing the correct sign of tropical Pacific SST gradient changes lies in the sparseness of data and differences between observational datasets, which apply varying methods to correct observational biases, especially during the World War II period and pre-1880^9,13^. The uncertainties in SST measurements also hamper our understanding of the link between changes in ENSO and mean tropical SST on decadal to centennial scales^14,15^. Several ENSO reconstructions based on multi- or univariate proxy archives have provided valuable insights into the past behavior of ENSO and underlying centennial and decadal mean state changes^16–23^. However, ENSO reconstructions do not fully agree on El Niño or La Niña mean states and partly consider either the center of action in the Niño3.4 domain, the distinction between eastern (EP) and central Pacific (CP) ENSO patterns, and teleconnection regions^22,23^.

**Figure 1:**
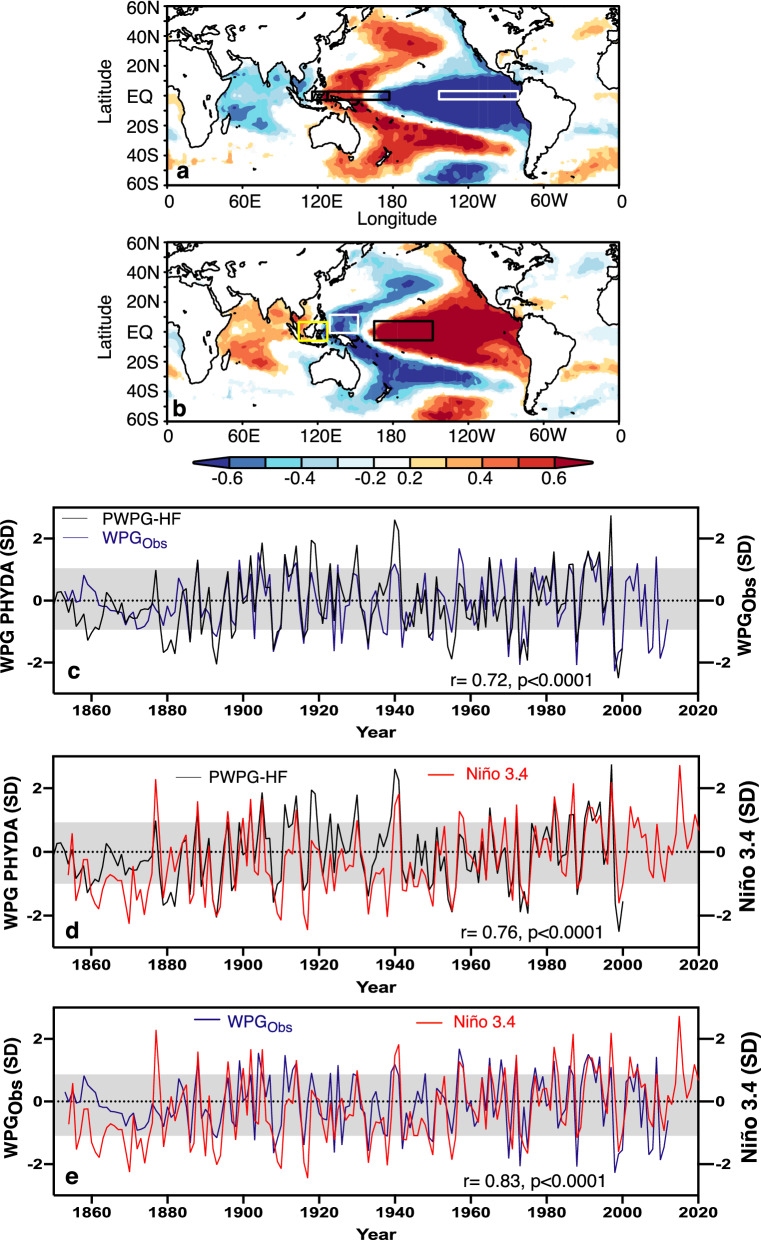
Zonal Pacific SST gradient (COBE^24^) and West Pacific Gradient (WPG^24^) correlation with HadISST^14^ and WPG PHYDA reconstruction^34^. Time series were normalised relative to 1961–1990 (SD = standard deviation). (**a**) Zonal SST gradient from COBE2^57^ following definition of Coats and Karnauskas^9^ (SST difference between 2.5°N–S, 117°E–173°E and 2.5°N–S, 205°E–275°E; see rectangular boxes) correlated with HadISST and (**b**) WPG following definition of Hoell and Funk^24^ correlated with HadISST (black and white box = WPG^24^; yellow and black box = WPG^25^). Spatial correlations in (**a**) and (**b**) computed in KNMI climate explorer^61^ (https://climexp.knmi.nl/), only correlation at 95% level coloured. PHYDA-West Pacific Gradient (WPG) reconstruction with 1 SD (grey bar) compared with (**c**) observed WPG after^24^ based on ERSSTv3b and (**d**) observed Niño3.4 index^2^ and (**e**) observed WPG after^24^ based on ERSSTv3b and observed Niño3.4 index^2^. Correlation coefficients (year to year) are indicated over full period of overlap for detrended data.

Since small changes in Pacific SST gradients connected with ENSO are related to global climate anomalies, it is of paramount importance to develop robust indices of their past behavior. Research has shown that the temperature difference between the westernmost Pacific and the central tropical Pacific region (hereafter West Pacific Gradient or WPG) plays a pivotal role in the global climate teleconnections^24,25^. The WPG is defined as the standardized difference between the central Pacific (Niño4 region; 5°S–5°N, 160–210°E) and western Pacific SST (0–10°N, 130–150°E) between 1854 and present from ERSSTv3b^56^. Hoell and Funk^24^ and Hoell et al.^26,27^ demonstrated that during both El Niño and La Niña events, the global impacts in terms of atmospheric circulation and precipitation anomalies were larger when the SST anomalies in the western Pacific were strongly opposing those in the central Pacific than when the western Pacific SST anomalies were near neutral. These studies show that the SST gradient between the central (Niño4 region) and the western Pacific was an essential measure of interannual to multidecadal Pacific climate variability in addition to any previously derived metric of ENSO^2^ or combination of ENSO metrics^24,28^. Furthermore, the spatial correlation patterns between the WPG with global air temperatures and SLP grid points resemble the pattern observed using the zonal (West minus East) Pacific SST and SLP gradient used to define the PWC (Fig. 1).

Recent changes in the WPG due to strong western Pacific warming and more frequent La Niña events after the Indo-Pacific decadal climate regime shift of the late 1990s to its La Niña-like phase^29–31^, are driving significant thermal anomalies impacting downstream coral reef ecosystems over several thousands of kilometers from the Indonesian seas to the southern coast of Western Australia and along the southwest Pacific. The abrupt rise in western Pacific SST in the late 1990s was also addressed by recent studies^14,15^. Hoell and Funk^24^ showed that the abrupt warming of the western Pacific has resulted in a more negative WPG, which has forced strong drought-inducing teleconnections across the Northern Hemisphere and the circum-Indian Ocean. Furthermore, Funk and Hoell^32^ showed evidence for a western Pacific warming mode with a V-shaped SST pattern radiating from the Maritime Continent towards the extratropics. This SST pattern tracks anthropogenic radiative forcing and can lead to drought-inducing atmospheric circulation changes. Recently, Cai et al.^25^ showed, for a subset of CMIP5 models, a high likelihood of a more intense WPG between the Maritime continent and the central Pacific (Niño4 region) and intensified La Niña events in 21st-century projections with drought-inducing teleconnections. Cai et al.^25^ further highlighted the importance of the WPG in modulating PWC strength by shifting SST patterns, ultimately also shifting atmospheric convection centers and rainfall.

Consequently, this study aims to reconstruct the WPG for the past Millennium to draw novel insights into past tropical climate variability. We make use of the Last Millennium reconstruction from PHYDA based on the Past Global Changes (PAGES2K) data archive for the Common Era^33,34^, which contains a multivariate proxy data array to assess the interannual and decadal variability in the WPG, Maritime Continent temperatures, the Niño4 and Niño3.4 regions, and the zonal SST gradient at annual resolution since 1000 CE. We will demonstrate that the WPG is a robust index that tracks the zonal tropical Pacific SST and SLP gradients, thus the PWC, and associated climate teleconnections. Our results reveal distinct periods of persistent El Niño or La Niña-like conditions during the past Millennium associated with shifts in the strength of the PWC and Maritime Continent temperatures affecting global climate and drought occurrences.

## Results

**Figure 2:**
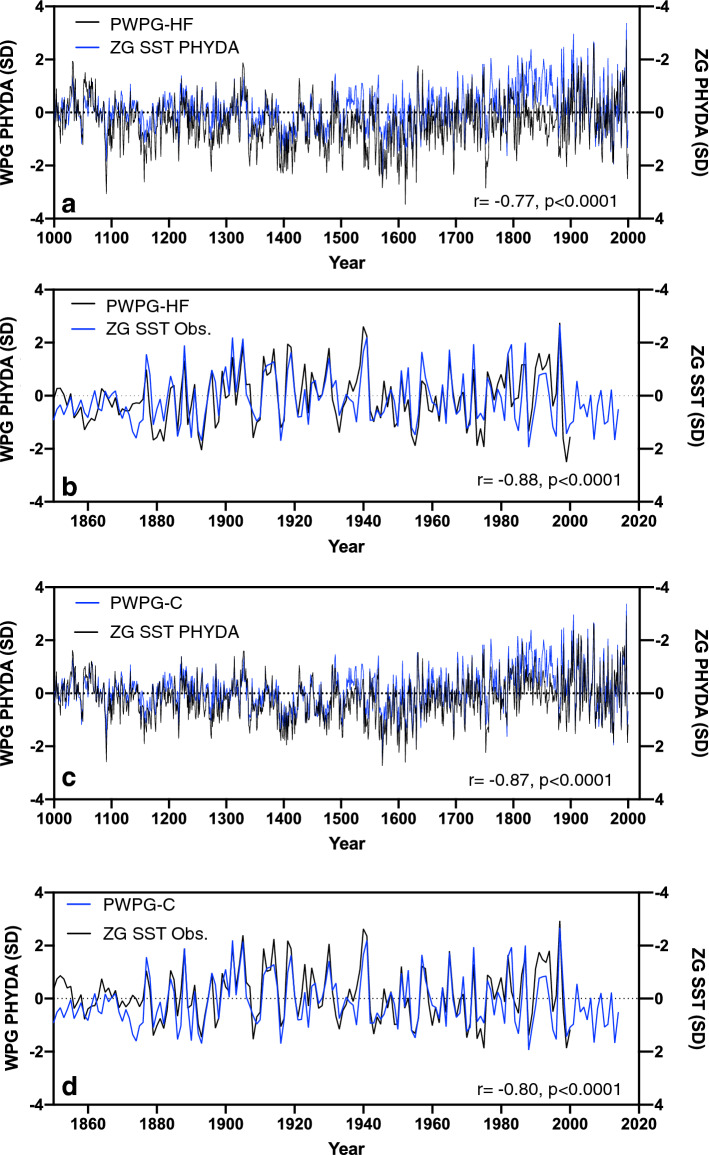
Reconstructed and Observed zonal (West–East) Pacific SST Gradient (ZG) compared to West Pacific Gradient (WPG) reconstructions. Time series were normalised relative to 1961–1990 (SD = standard deviation). PWPG-HF reconstruction compared with (**a**) PHYDA zonal SST gradient after Coats and Karnauskas^9^, (**b**) observed zonal SST gradient from COBE2 SST (axis inverted^57^) after Coats and Karnauskas^9^. PWPG-C reconstruction compared with (**c**) PHYDA zonal SST gradient after Coats and Karnauskas^9^ and (**d**) observed zonal SST gradient after Coats and Karnauskas^9^ from COBE2 SST (axis inverted^57^). Correlation coefficients (year to year) are indicated over full period of overlap for detrended data.

**Figure 3:**
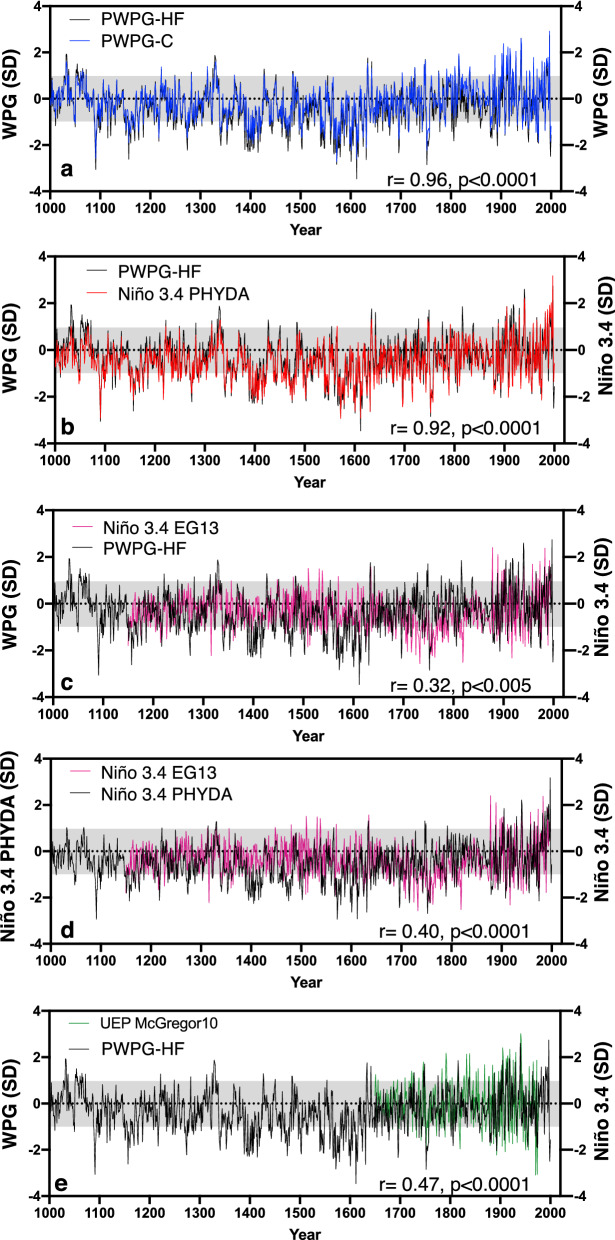
Mean annual reconstruction of the WPG from the Last Millennium PHYDA^34^ multivariate proxy network. Time series were normalised relative to 1961–1990 (SD = standard deviation). WPG based on Hoell and Funk^24^ (black line) with one standard deviation (SD; grey bar) threshold compared to (**a**) WPG based on definition of Cai et al.^25^ (blue), both calculated from PHYDA, (**b**) PHYDA-based Niño3.4 index (red line), (**c**) Niño3.4 reconstruction (magenta line; EG13) of Emile-Geay et al.^22^, (**d**) ^34^PHYDA-based Niño3.4 index (black line) compared to Niño3.4 reconstruction (magenta line; EG13) of Emile-Geay et al.^22^ and (**e**) the WPG based on Hoell and Funk^24^ (black line) compared to the Niño3.4 reconstruction (green line; McGregor10) of McGregor et al.^18^. Correlation coefficients (year to year) are indicated over full period of overlap for detrended data.

The spatial pattern of the tropical Pacific SST field is characterized by a zonal SST gradient (Fig. 1a) which strongly resembles the spatial SST pattern related to the WPG based on ERSST (Fig. 1b)^24^. Results for the observed WPG based on the definition by Cai et al.^25^ are similar. We, therefore, focus on the Hoell and Funk^24^ WPG (hereafter WPG_obs_) for most of the study. The typical horseshoe pattern in SST related to ENSO emerges in both spatial correlations. Our reconstructed PHYDA-derived WPG based on the definitions by Hoell and Funk^24^ (hereafter PWPG-HF; see Fig. S1 for WPG^25^) revealed close agreement in reconstructed amplitude variations with the Niño3.4 index^2^ based on observational data from 1854 onwards (Fig. 1c-e; Fig. S1). We find a significant correlation between WPG_obs_ and Niño3.4 indices with our reconstructed WPG indices between 1854 and 2000 (Figs. S2 and S3). The PWPG-HF shows higher amplitude variations than the observational WPG and the Niño3.4 index (Fig. 1d) which also holds for the WPG_obs_ and PHYDA WPG Cai (hereafter PWPG-C; Fig. 1e; Fig. S1). We also identified co-variability between the zonal Pacific SST (hereafter ZG_sst_) and SLP (hereafter ZG_slp_) gradients (definitions of^9,10,35^) reflecting the Walker Circulation with both PHYDA-WPG reconstructions (Fig. 2; Fig. S4). In general, we observed a stable relationship with WPG_obs_, Niño3.4, ZG_sst_, and ZG_slp_ between 1880 and 2000 with the lowest correlations pre-1880 (Fig. S2). The latter is most likely due to sparse observations pre-1880 with higher uncertainties in instrumental data (results for PWPG-C are similar, see Fig. S2). The WPG_obs_, Niño3.4, and ZG_sst_ are consistent with each other showing stable relationships throughout the record with a breakdown pre-1880 (Figs. 2, 3; Figs. S2 and S3). The WPG_obs_ also displayed a stable relationship with the zonal SLP gradient mirroring the relationship shown by the PHYDA WPG’s (Fig. S5). Other ENSO reconstructions display significant relationships with the ZG_sst_ and ZG_slp_, yet less stable than both PHYDA WPGs (Fig. S6).

Having established that the reconstructed PWPG-HF and PWPG-C reflect stable relationships for the instrumental data period, we now consider the full Last Millennium reconstruction of the WPG and Niño3.4. Both PHYDA WPGs are nearly identical with slight amplitude differences (Figs. 2a,c; 3a; Fig. S7). Both WPG reconstructions also co-vary with the ZG_sst_ and Niño3.4 from PHYDA, again with slight differences in absolute magnitudes (Figs. 2a,c, 3b; Fig. S7). There is no long-term trend in both WPGs, yet significant interannual to multidecadal variability (Figs. 2, 3). Most of the eleventh century was dominated by a positive WPG and Niño3.4 (negative ZG_sst_), switching to negative WPG and Niño3.4 (positive ZG_sst_) towards the late eleventh century. Between 1100 and 1300 CE, neutral or weak positive WPG conditions alternated with moderate to strong negative WPG. The early fourteenth century experienced strong positive WPGs followed by strong negative WPGs (positive ZG_sst_) in the mid fourteenth century and around 1400 CE. Between 1400 and 1600 CE, negative WPG and Niño3.4 anomalies (positive ZG_sst_) dominated with intermittent weak to moderate positive WPG periods (negative ZG_sst_). The early seventeenth century have seen individual strong positive WPG (negative ZG_sst_) years followed by a period of weakly positive WPG years alternating with moderate to strongly negative WPG conditions. The mid-eighteenth century showed a brief period of extreme WPG swings from strongly positive to strongly negative WPG (similar for ZG_sst_). During the early to mid-eighteenth century, the ZG_sst_ agrees better with PWPG-C than with PWPG-HF (Fig. 2a, c) with the ZG_sst_ (positive PWPG-C) on average in a negative mean state, while PWPG-HF shows only moderate swings from positive to negative. The early and late nineteenth century experienced strong negative WPGs (positive ZG_sst_), while the early and late twentieth century was dominated by both strong positive and negative WPG conditions (similar for ZG_sst_) with weaker variability in the mid-twentieth century.

Both reconstructed WPGs also compared well with palaeo-Niño3.4 indices by Emile-Geay et al.^22,23^ (hereafter Niño3.4 EG13) starting in 1150 CE and McGregor et al.^18^ (hereafter MG10; Fig. 3c, e; Figs. S8 and S10) starting in 1650 CE. The palaeo-Niño3.4 indices by Emile-Geay et al.^22,23^ also co-varies with the PHYDA Niño3.4 based reconstruction (Fig. 3d; Figs. S7 and S10). However, differences in absolute magnitudes and signs were observed with the palaeo-Niño3.4 EG13, for instance, around 1150 CE, 1400 CE, and during the late sixteenth, early seventeenth, and eighteenth centuries. With MG10, the interannual and decadal variability is well-matched to both PHYDA WPGs, yet the absolute magnitude was higher in MG10 for individual years or events (Fig. 3e; Figs. S7, S8 and S10). 31-year running correlations confirmed the stability of relationships between our PHYDA WPGs and Niño3.4 reconstructions with the palaeo-Niño3.4 EG13 and MG10 indices for most of the record, although several multidecadal periods show weaker correlations (Fig. 2; Figs. S7, S8 and S10). Interestingly, one of those periods with weaker correlations was around the mid-nineteenth century. Other periods with weaker relationships include the 13th, 15th, and late eighteenth centuries (Fig. 3; Figs. S7 and 8). We also compared our WPGs with a recent reconstruction of ENSO flavors from coral proxy data^47^, extending back to 1620 CE to consider temporally shifting centers of action for ENSO (Fig. S9). We found tight relationships during the twentieth century with both the Niño cold tongue (NCT) and Niño warm pool (NWP) indices (Fig. S9), generally stronger with the NCT. The early and mid-nineteenth centuries showed weakening correlations with both NCT and NWP. Correlations with the NWP were strong around 1750 CE, while those with the NCT were weak. Around 1700 CE the NCT (NWP) showed stronger relationships (weaker) with both PHYDA WPG's, while pre-1700 CE, both NWP and NCT showed weak to moderately strong relationships (Fig. S9). We also assessed the relationship of the paleo-Niño3.4 EG13 and MG10 with observed Niño3.4, as well as the zonal SST and SLP indices (Fig. S10). MG10 and Niño3.4 EG13 indicate relatively stable relationships with observed Niño3.4 and zonal SST/SLP gradients pre-1960 with a weakening post-1960 (Fig. S5) while the PHYDA Niño3.4 reconstruction showed stable correlations for the entire record (Figs. S2 and 3).

**Figure 4:**
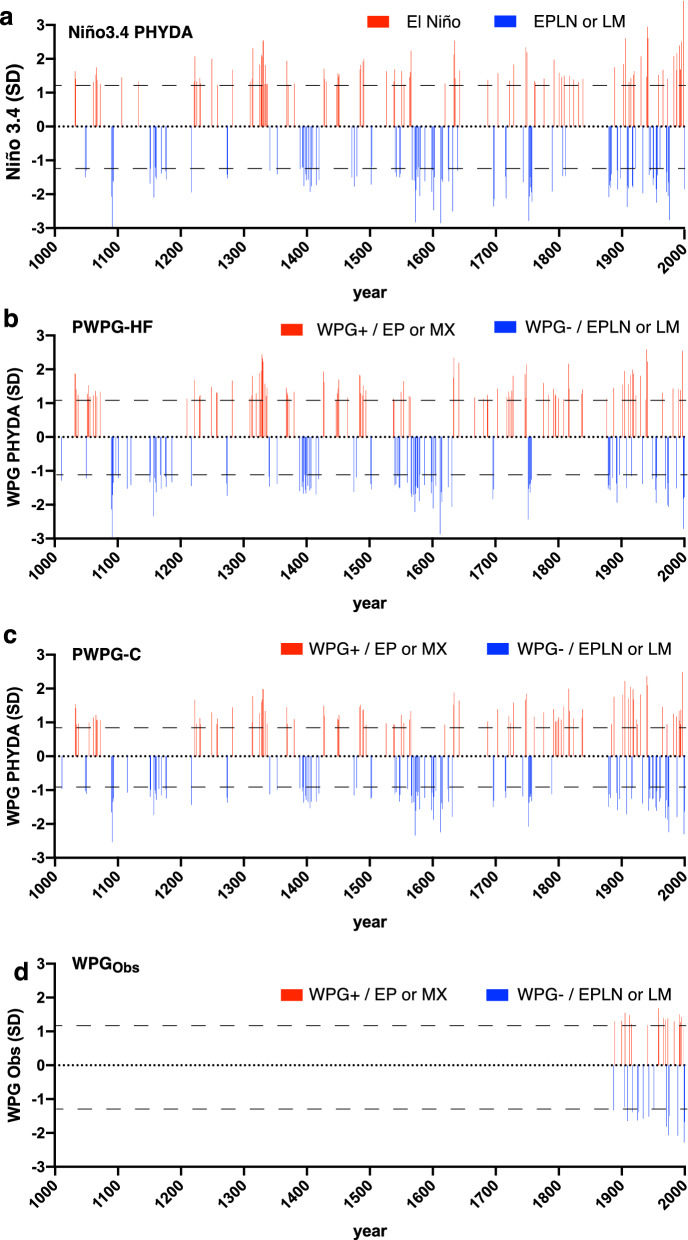
Extreme events based on 90th percentile of standardized and detrended indices. Time series were normalised relative to 1961–1990 (SD = standard deviation). (**a**) Niño3.4 index from PHYDA^34^, (**b**) PHYDA-WPG (PWPG-HF) based on Hoell and Funk^24^, (**c**) PHYDA-WPG (PWPG-C) based on Cai et al.^25^, and (**d**) observed WPG based on Hoell and Funk^24^. All positive (WPG +) and negative (WPG−) event thresholds > 90% percentile unique to each index illustrated by dashed lines. Only events that exceed the 90th percentile are shown. EP = eastern Pacific El Niño events, MX = mixed eastern-central Pacific El Niño events, EPLN = eastern Pacific La Niña, LM = La Niña Modoki-type events.

In order to extract the occurrence of extreme positive WPG and negative WPG periods across the last Millennium, we grouped the extreme events, calculated as the 90th percentile of each index, in our reconstructions and the observed WPG (Fig. 4; Table 1). This analysis revealed the co-occurrence of extreme negative WPG and Niño3.4 anomalies during the early and late twelfth century, around 1400 CE and between the fourteenth and sixteenth centuries and several other shorter periods, as does the co-occurrence of extreme positive WPG and Niño3.4 anomalies in the early eleventh century and the twentieth century. The late sixteenth to early seventeenth century was characterized by a warmer western than central Pacific with multiple high-magnitude negative WPGs and Niño3.4 years. The Medieval Climate Anomaly (1100–1350 CE) was dominated by multidecadal swings of positive and negative WPG and Niño3.4 anomalies with a slight preference for higher amplitude negative WPG conditions compared to fewer high magnitude positive WPG. This agrees with Goodwin et al.^36,37^, who found a multidecadal dominance of EP La Niña and CP La Niña during the MCA. During the colder eighteenth and nineteenth centuries in the western Pacific, the occurrence between positive and negative WPG was more balanced with a dominance of interannual variability. The twentieth century indicated an unprecedented cluster of frequent co-occurrences of extreme negative WPG and Niño3.4 and extreme positive WPG and Niño3.4 anomalies. We assessed changes in WPG variability through 31-year running standard deviations in both PHYDA WPGs and Niño3.4 from PHYDA and Niño3.4 EG13 over the entire length of records (Fig. S11). The twentieth century showed the most extreme variability over the Last Millennium with equally strong positive and negative WPG and Niño3.4 anomalies (Fig. 4; Fig. S11). However, the diminishing quantity of proxy data in the early part of the Last Millennium calls for caution when inferring changes in variance between the modern period and the distant past. Nevertheless, the Niño3.4 EG13 index mostly agrees with our results based on WPG reconstructions in showing the highest variability in the twentieth century and enhanced standard deviations in the early 13th, sixteenth, and early seventeenth centuries (Fig. S11).

**Table 1:**
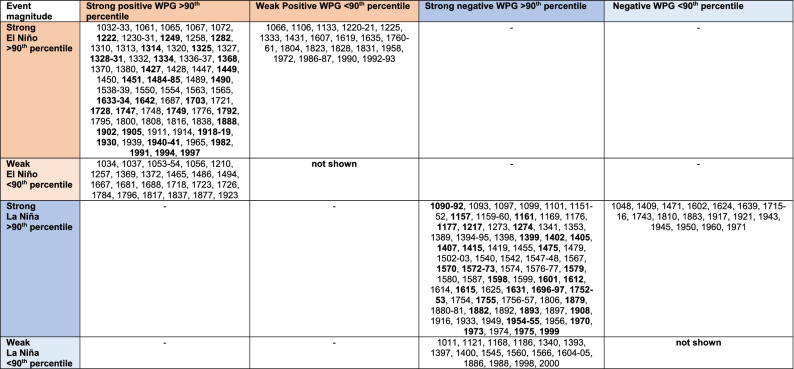
Extreme events in PHYDA based on 90th percentile of normalised and detrended indices illustrated in Fig. 4. Time series were normalised relative to 1961–1990. Simultaneous extreme events in WPG and Niño3.4 indices based on the 95th percentile marked in bold letters. Negative WPG events <90th percentile co-occurring with strong La Niña are also indicated. Weak positive WPG events < 90th percentile co-occurring with strong El Niño events in the PHYDA reconstruction are also indicated.

**Figure 5:**
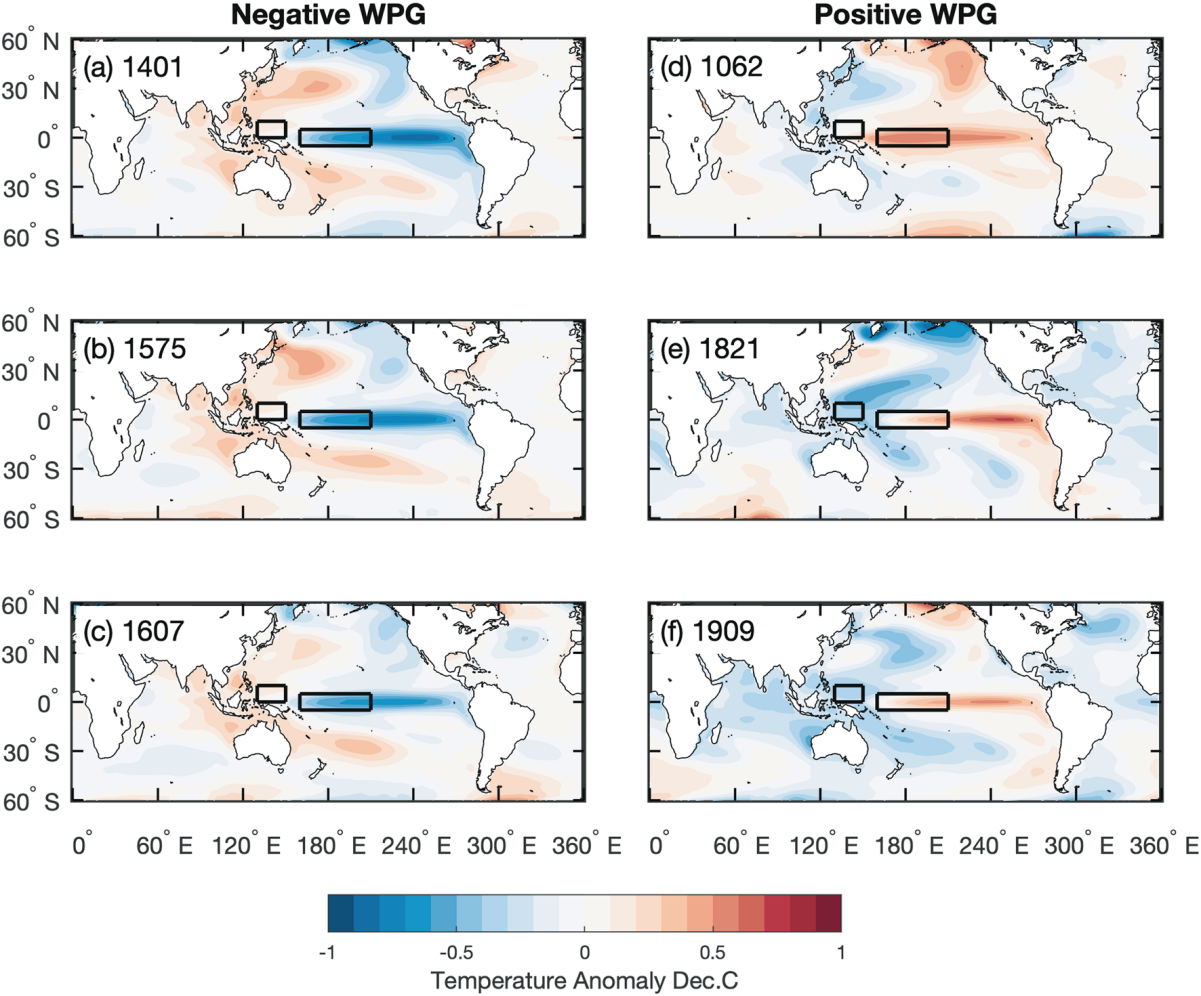
Composites of 20 year mean sea surface temperature anomalies (30°N–30°S, 100°E–75°W) in ^34^PHYDA^46^ over the past Millennium. The 20-year periods were chosen as the strongest negative (**a**-**c**) and positive (**d**-**f**) anomalies in the WGP timeseries and the anomaly is calculated relative to the 1001–2000 mean. The 20-year period is centred on the indicated year: for example, 1401 is the mean of 1392 to 1411. Location of the WPG grid boxes after Hoell and Funk^24^ indicated by black rectangles. Figures created in Matlab R2020b (https://au.mathworks.com).

**Figure 6:**
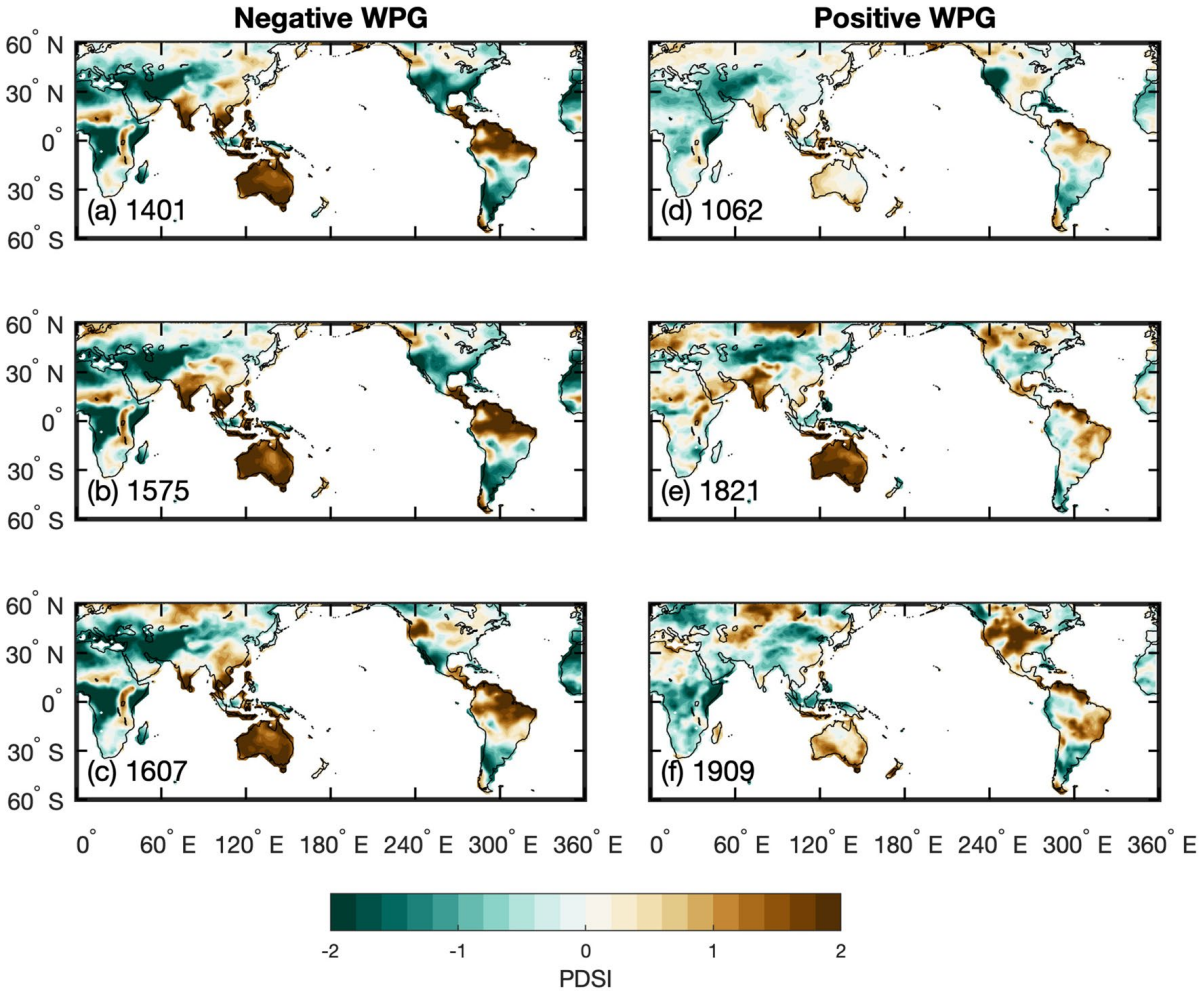
Composites of 20-year mean anomalies of the spatial Palmer Drought Severity Index in ^34^PHYDA (PDSI; 30°N–30°S, 100°E–75°W) for the most extreme 20-year periods of (**a**-**c**) negative and (**d**-**f**) positive West Pacific Gradient (WPG)^46^ over the past Millennium. The anomaly is calculated relative to the 1001–2000 mean. The composites were based on the SST patterns shown in Fig. 5. The 20-year period is centred on the indicated year: for example, 1401 is the mean of 1392 to 1411. Location of the WPG grid boxes after Hoell and Funk^24^ indicated by black rectangles. Figures created in Matlab R2020b (https://au.mathworks.com).

To illustrate the spatial SST and PDSI patterns during extreme WPG swings, we computed 20-year mean anomalies for the most extreme negative and positive multidecadal WPG periods over the past Millennium (Figs. 5, 6). Negative WPG extremes all resemble La Niña-like SST patterns (Figs. 5a-c; 6a-c). Negative WPG extremes at the turn of the 14th to the fifteenth centuries and the sixteenth to the seventeenth centuries share similar SST spatial patterns with maximum cooling in the central (Niño4 region) and eastern Pacific (Niño3.4 region). The positive WPG extremes appear more variable in their spatial SST footprint, though all these extremes resemble an El Niño-like SST pattern (Figs. 5a-c; 6a-c). In the eleventh century, the center of maximum warming was located in the central Pacific (Niño4 region), while in the early nineteenth and early twentieth century, maximum warming was found in the eastern Pacific (Niño3.4 region).

**Figure 7:**
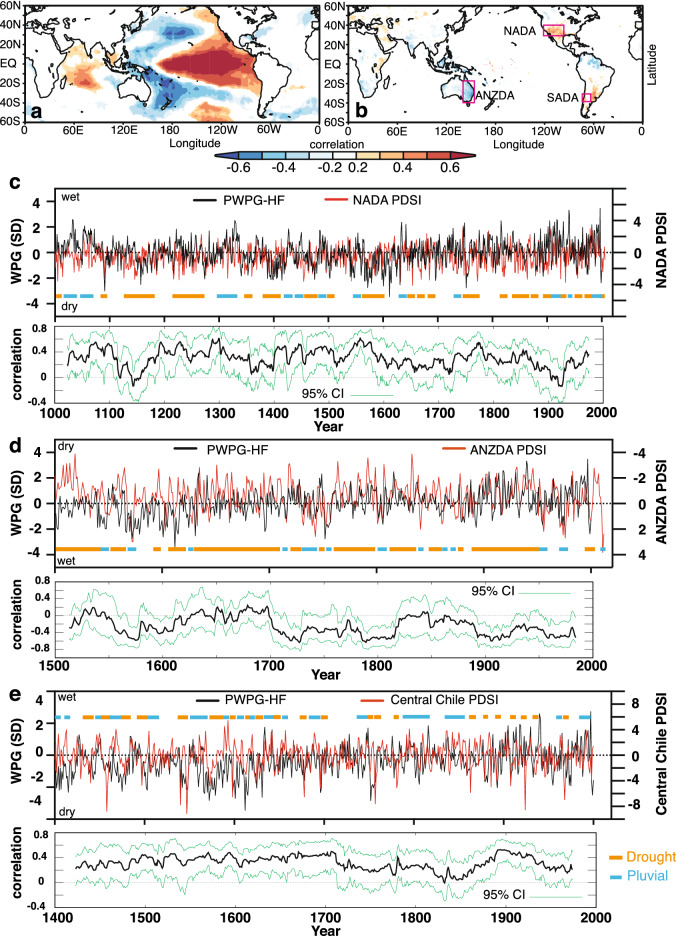
Comparison between the West Pacific Gradient (WPG) reconstruction from PHYDA^34^ and palaeohydrological data. Time series were normalised relative to 1961–1990 (SD = standard deviation). (**a**) Spatial correlations between the PHYDA-West Pacific Gradient (PWPG-HF) reconstruction (after^24^) with instrumental ^58^HadISST and (**b**) with GPCC rainfall^59^. Only correlation > 95% significance coloured. Spatial correlations in (**a**) and (**b**) computed in KNMI climate explorer^61^ (https://climexp.knmi.nl/). PHYDA-WPG (PWPG-HF) compared to (**c**) North American drought atlas (30–40°N, 125–105°W^62^) and 51-year running correlation, (**d**) Australia-Nea Zealand drought atlas PDSI (18–29°S, 140–155°E; ANZDA^38^) and 51-year running correlation, and (**e**) Central Chile PDSI from the South America Drought Atlas (31–37°S, 70–72°W; SADA^42^) and 51-year running correlation. Dry (orange) and wet (blue) conditions are indicated. Correlation coefficients (year to year) are also indicated over full period of overlap for detrended data.

**Figure 8:**
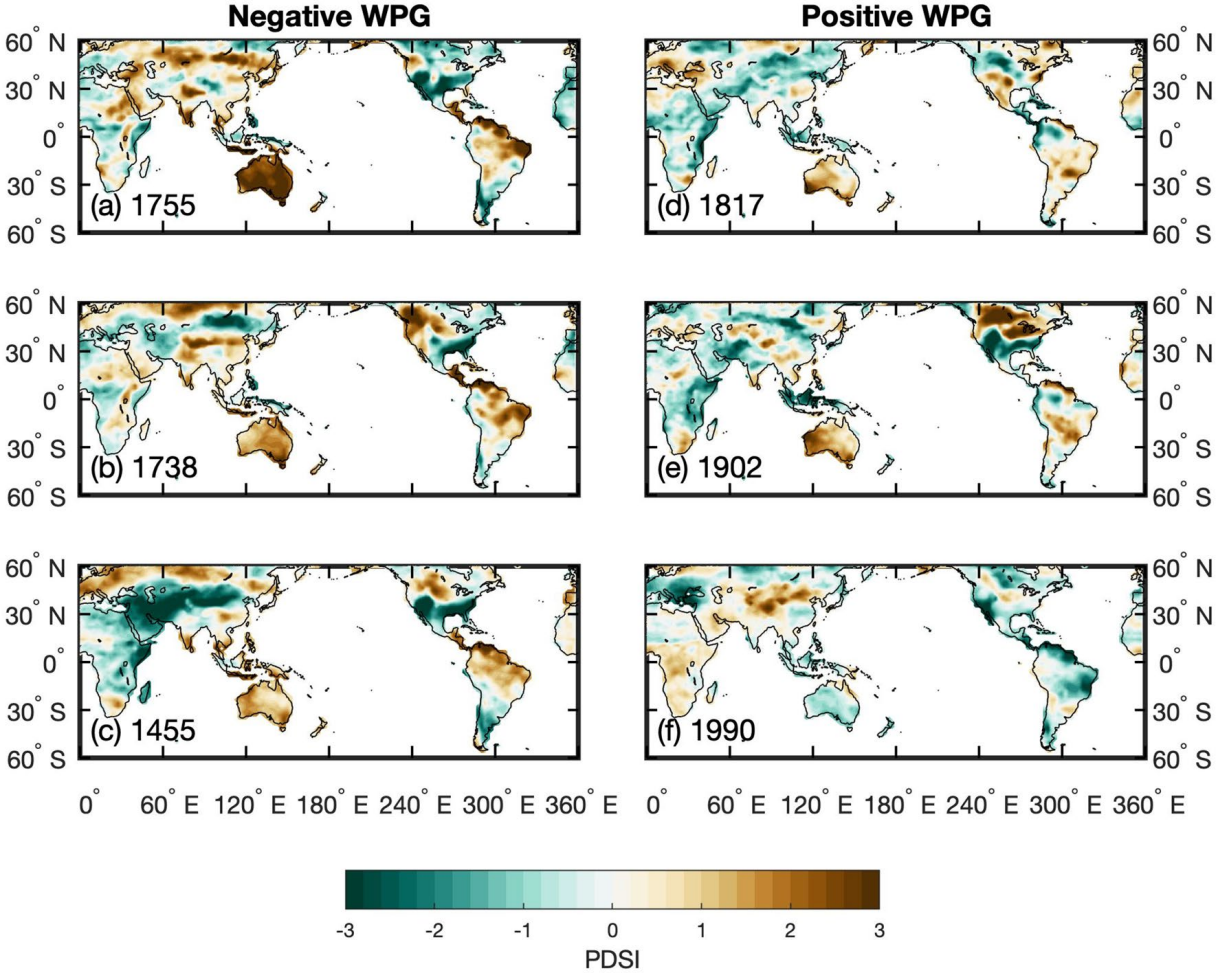
Yearly mean anomalies (April to March) of the spatial Palmer Drought Severity Index in ^34^PHYDA (PDSI) for selected time periods with left) negative and right) positive West Pacific Gradient (WPG). (**a**) strong negative WPG associated with strong La Niña, (**b**) strong negative WPG associated with weak La Niña and (**c**) strong La Niña associated with weak negative WPG. (**d**) strong positive WPG associated with weak El Niño, (**e**) strong positive WPG associated with strong El Niño and (**f**) weak positive WPG associated with strong El Niño. Figures created in Matlab R2020b (https://au.mathworks.com).

The question arises if changes in the spatial Pacific SST pattern variability over the past Millennium had influenced rainfall-drought cycles in global hydrology. To answer this question, we first verified if the reconstructed WPG does reproduce the observed relationships with SST, SLP, and rainfall across known ENSO impacted regions since 1850 CE (Figs. S2, S3, S5). Figure 7 illustrates that our PWPG-HF index reproduced the expected spatial correlations, as does the observed WPG. The strongest relationships were found between SST and rainfall across the Indo-Pacific Ocean and rainfall in Eastern Australia, the Maritime Continent, the Indian Monsoon region, southwest North America, and the rainfall dipoles between North and South America and East and South Africa. Subsequently, we have contrasted our WPG time series with those of long-term hydrological reconstructions. We used the drought atlases for Australia and New Zealand (ANZDA^38^), the North American Drought Atlas (NADA^39–41^), and South American Drought Atlas (SADA^42^) as examples, next to the PHYDA-based PDSI field reconstruction (Figs. 6, 7c-e; Figs. S12). Figure 7 illustrates that our PWPG-HF index co-varied positively with the NADA for southwestern North America and SADA grid boxes for central Chile, while it is negative with the ANZDA grid box for eastern Australia. The multidecadal oscillation of dry and wet periods observed in NADA between 1200 and 1600 CE and 1850 CE to present was mirrored by the WPG indices (Fig. 7c). The extreme multidecadal NADA droughts in southwestern North America around 1400 CE and in the late sixteenth to early seventeenth century were clearly associated with a series of extreme negative WPG and Niño3.4 anomalies (Fig. 7c). The ANZDA time series revealed that positive WPG and Niño3.4 conditions were associated with dry anomalies in eastern Australia and vice versa for wet periods (Fig. 7d). The South American record from central Chile displayed an association of wet periods with positive WPG and Niño3.4 and vice versa for dry periods (Fig. 7e). Again, the late sixteenth century indicated dry anomalies with strong negative WPGs and Niño3.4 anomalies. The spatial PDSI pattern in PHYDA for the most extreme 20-year negative WPG periods agrees with our results based on individual PDSI reconstructions (Figs. 5–7; Figs. S12-13). On the contrary, the most extreme 20-year positive WPG periods illustrated in Figs. 5 and 6 show a more variable response, most probably related to the magnitude of individual positive WPG phases and their specific spatial SST pattern. For instance, the most extreme period centered around 1909 indicates the expected wet conditions in southwestern North America and central parts of South America, while the Maritime Continent and southeast Asia show dry conditions. Thus, the magnitude of the WPG during ENSO and the resulting SST pattern across the tropical Pacific appears to influence the global rainfall teleconnections. To further illustrate the role of the WPG in global rainfall teleconnections, we extracted the spatial PDSI patterns for years with strong positive (negative) WPG co-occurring with strong or weak El Niño (La Niña), and years where a strong El Niño (La Niña) was associated with a weak positive (negative) WPG in ^34^PHYDA (Table 1; Fig. 8; Fig. S14). For example, 1755 CE (strong negative WPG, strong La Niña) differs from 1455 CE (strong La Niña, weak negative WPG) in terms of PDSI anomalies across Asia and Africa, as well as eastern Australia and southeast Asia (Fig. 8a,c). 1902 CE (strong El Niño, strong positive WPG) reveals stronger drought anomalies across southeast Asia then 1817 CE (weak El Niño, strong positive WPG; Fig. 8d,e), while the response over North America differs from expectations in 1902 CE. In 1902 CE, the spatial SST pattern indicates warming in the northern and southern Atlantic which is not observed in 1817 CE (Fig. S14). 1990 CE (weak positive WPG, strong El Niño; Fig. 8f.) shows a global dry PDSI pattern with opposite signals in eastern/southern Africa and across central Asia that may also be influenced by a global warming signature (Fig. S14). Thus, the PDSI pattern differs substantially between the selected cases, highlighting the role of Pacific and potentially pan-oceanic SST patterns in modulating far-field rainfall and drought responses.

### Discussion

We have shown that the WPG during ENSO is a robust index for Last Millennium Walker Circulation variability, the latter defined by SST and SLP gradient indices across the tropical Pacific^4,8–10^. For the twentieth century, we also show that the WPG captured the SST and precipitation teleconnection patterns qualitatively similar to ENSO and Walker Circulation indices. Compared to other paleo-ENSO indices^18,22,23^, the WPG indicated a more stable relationship with the observed PWC indices^9^. Thus, the WPG is a crucial index for the PWC in the instrumental era and the past Millennium.

We did not observe a clear linear trend in the WPG during the Last Millennium. Instead, both Last Millennium WPGs and ZG_sst_ showed multidecadal to multi-centennial variability with more negative WPG and canonical La Niña or CP La Niña-like occurrences (positive ZG_sst_) in the late 11th, mid-late 12th, at the turn of the fourteenth century, between the fourteenth to sixteenth, mid-seventeenth centuries and since 1870 CE (West Pacific warmer than the central Pacific). Positive WPG and canonical El Niño or CP El Niño-like occurrences (negative ZG_sst_) dominated the first century of the Last Millennium, the early 14th and short periods in the eleventh and eighteenth centuries (West Pacific colder than central Pacific). The twentieth century stands out as a period with both strong positive and negative WPG (and ZG_sst_) occurrences and overall highest variability over the past Millennium. However, several studies caution oversimplified interpretations of variance changes due to diminishing proxy availability over time^43,44^. Nevertheless, our results agree with other Niño3.4 indices in terms of multidecadal oscillations and changing variance over time, despite vastly different methodologies^8,22,45,46^.

Arguably, the WPG could be an excellent index for CP ENSO or Modoki phases which often are characterized by strong SST gradients between the western and central Pacific, yet the same sign anomalies in the eastern and for the western Pacific. Hoell and Funk^24^ showed evidence that most negative WPG phases since 1950 were associated with central Pacific or La Niña Modoki events, while positive WPG phases had an affinity to occur predominantly during eastern Pacific or canonical El Niño events. However, several positive and negative WPG phases were associated with mixed-type ENSO flavors. Thus, periods of extreme negative WPG in our Last Millennium reconstructions may represent canonical La Niña or La Niña Modoki conditions, while positive WPG periods may indicate canonical El Niño or mixed central and eastern Pacific El Niño flavors. We turned to the Niño Cold Tongue (NCT) and Niño Warm Pool (NWP) reconstructions based on Pacific coral proxy records to assess their relationship with the WPG^47^ (Figs. 3 and S8). The NWP and NCT study considered El Niño flavors only, yet the index also includes the La Niña flavors. We hypothesized that the NWP should predominantly co-vary with the WPG positive (negative) events when the latter would predominantly reflect Modoki or central Pacific El Niño (central Pacific La Niña) events. The comparison of the WPG indices with the NCT and NWP ENSO flavor indices revealed time-varying relationships, especially between 1600 and 1850 CE, yet the strongest co-variability with both since the mid-nineteenth century to the present^47^ (Fig. S8). During the mid/end-eighteenth century, the relationship revealed a closer agreement with the NWP (central Pacific flavor), while the early eighteenth-century WPG indicated closer alignment with the NCT (eastern Pacific flavors). However, the eighteenth century is also known for its reduced proxy data coverage in the Pacific Ocean, so it should be viewed with caution^47^. The enhanced central to eastern Pacific event ratio during the seventeenth century is less clearly reflected by the WPG’s. The WPG’s do, however, indicated extreme negative events (positive ZG_sst_) in the early and late seventeenth century (Figs. 2, 3). However, when taking into account centennial variability (without detrending), the WPG appears to reflect dominantly negative WPG or La Niña Modoki conditions except for the eleventh and twentieth centuries, the early 14th and early eighteenth centuries, and several short-lived periods over the past Millennium (Fig. 2). Composites for the most extreme 20-year WPG periods in ^34^PHYDA since 1000 CE confirmed that the negative WPG extremes show less variable spatial SST patterns, thus often resemble canonical La Niña or La Niña Modoki conditions with an increased ZG_sst_. On the contrary, positive WPG extremes resemble mixed central and eastern Pacific El Niño flavors with reduced ZG_sst_. Thus, there appears to be a relationship between the Pacific SST mean state, ZG_sst_, and the WPG.

Hoell and Funk^24^ and Hoell et al.^26,27^ demonstrated how the magnitude of the WPG during the instrumental data period (1948–2011) modifies atmospheric circulation anomalies, thereby modulating the rainfall anomalies across the Indo-Pacific during strong El Niño and La Niña years (see Figs. 9 and 10 in^24^). Here, we established the hydroclimatic relationships of the WPG with the three drought atlases in the preindustrial period, which were mainly in agreement with assessments of preindustrial ENSO impacts on drought and pluvial conditions. Following the findings of Steiger et al.^46^, megadroughts in southwestern North America were found to be associated with colder values of the Niño3.4 index and an extremely negative WPG (positive ZG_sst_) in PHYDA. The North American megadroughts around 1400 CE and the sixteenth to seventeenth century turn during the Little Ice Age stand out as periods with persistent negative WPG and Niño3.4 anomalies (positive ZG_sst_). The latter is consistent with Dee et al.^44^ and Steiger et al.^46^, who showed evidence for enhanced North America ENSO teleconnections during the Little Ice Age. The latter held for both El Niño and La Niña events and was unaffected by proxy availability over time. In other words, we find more drying in the North American Southwest in more negative WPG years (positive ZG_sst_) during periods of enhanced ENSO teleconnections. Steiger et al.^46^ pointed out that the megadroughts pre-1600 CE were likely radiatively unforced and most strongly associated with unusually cold SST in the central and eastern Pacific and warm SST in the Atlantic. Nevertheless, they also showed evidence for a potential role of local radiative forcing exacerbating regional drought in the North American Southwest. The western Pacific region of the WPG and the Maritime Continent are oceanic regions of high sensitivity to radiative forcing^11,12,32^. Thus, changes to the WPG might bear a footprint of local radiative forcing coupled with slow decadal oceanic feedbacks that affect the SST patterns in the Pacific and associated teleconnections^48^. The SST patterns across the western Pacific appear to play a crucial role in the drought and pluvial-inducing teleconnections in addition to those in the central and eastern Pacific. Thus, the megadrought around 1400 CE and the late sixteenth century was likely exacerbated by the strong WPG enhancing Pacific circulation anomalies, as observed for similar WPG-ENSO associations during the instrumental data period^24^. This adds credibility to the interpretation that unsual and extreme La Niña conditions during this time interval were likely drivers of the megadroughts.

Our analysis revealed that the WPG plays an important role in South American hydroclimate. The PHYDA WPG reconstructions revealed that wet conditions persisted in central Chile during a positive WPG while dry conditions prevailed during the negative WPG phases. This is essentially in agreement with ENSO teleconnections based on the SADA^42^. Our study suggested a weaker influence of the WPG on central Chile in parts of the eighteenth and nineteenth centuries. Here, influences from the Southern Annular Mode or Atlantic could have played a larger role^42^.

Eastern Australia showed wetter conditions during negative WPG (La Niña-like) phases, and drier conditions during positive WPG (El Niño-like) phases amplified by negative and positive Interdecadal Pacific Oscillation phases, respectively^38^. Our WPG reconstruction revealed three periods of negative correlations with the Eastern Australia region in ANZDA (higher rainfall) during the turn of the sixteenth to seventeenth century, between 1700–1800 CE and the twentieth century. Weaker relationships with the WPG were observed in between. The WPG also showed the typical anti-correlation between Eastern Australia and southern New Zealand PDSI in ANZDA between 1500 and 2000 CE^38^ (Fig. S13). The relationship during the twentieth century indicated that teleconnections with the WPG were strongest when ENSO variability was higher between 1880–1920 and since 1960. The latter is consistent with abundant evidence from previous studies of enhanced ENSO teleconnections during intense ENSO phases^49–51^. ENSO modulation by low-frequency oscillations of the Interdecadal Pacific Oscillation can modulate ENSO impacts on Australia^52,53^. The latter may also hold for relationships between Eastern Australia PDSI in ANZDA and the WPG.

In summary, the new PHYDA WPG reconstructions indicated robust hydroclimate teleconnections with regions sensitive to ENSO and WPG drought and pluvial conditions across the Pacific and on its rims. The late sixteenth century was confirmed as the period of strong negative WPG, positive ZG_sst_ and Niño3.4 occurrences with megadroughts in North America and South America and pluvials in Australia. This illustrates that the WPG may serve as a robust index together with ENSO indices to reveal the impact of drought-inducing climate teleconnections that arose from changes in Pacific SST patterns. We call for a concerted effort of generating new proxy data from this crucial region in the western Pacific which could dramatically improve the knowledge base for the centennial and decadal PWC changes.

## Methods

WPG temperature variability over the past Millennium is reconstructed using the Paleo Hydrodynamics Data Assimilation (PHYDA^34^; https://zenodo.org/record/1198817#.YGXM4khKjUI). PHYDA is analogous to modern reanalysis products but is constrained by paleoclimate data instead of meteorological observations^34,54^. PHYDA uses data assimilation of multivariate palaeoclimate proxy data with the dynamical constraints of a Last Millennial Ensemble simulation, the Community Earth System Model Ensemble (CESM LME^55^). The PHYDA approach preserves dynamical relationships between the ocean and atmospheric variables and accommodates periods of non-stationary teleconnections. PHYDA uses 2978 annually-resolved proxy records, which builds on the PAGES2k and a tree ring width proxy network^33^. In short, the DA reconstruction process computes the linear fit between the initial climate state (prior) based on the ^55^CESM LME and the proxies. PHYDA reconstructions of gridded 2 m temperature at 2° spatial grid resolution for annual means were accessed for this work. PHYDA derived climate parameters were extensively validated against instrumental observations in Steiger et al.^34^. We extracted all climate indices from PHYDA at annual resolution since 1000 CE. The PHYDA reconstruction of all variables includes the posterior ensemble mean and 1 standard deviation of the posterior ensemble. Thus, this error estimate takes into account the spread in the climate model prior of CESM LME as well as the error in the proxy models. For further details of the reconstruction methodology see Steiger et al.^34^.

The WPG temperature has been calculated using the Hoell and Funk^24^ and Cai et al.^25^ approach from PHYDA. The WPG was originally defined by Hoell and Funk^24^ as the standardized difference between the central Pacific (Niño4 region; 5°S–5°N, 160–210°E) and western Pacific SST (0–10°N, 130–150°E) between 1854 and 2010 from ERSSTv3b^56^. The WPG_Obs_ (based on Hoell and Funk^24^) from observations relies on a similar 2° spatial grid resolution as PHYDA. In addition, Cai et al.^25^ calculated an air temperature gradient between the Niño4 region (5°S–5°N, 160–210°E) and the Maritime continent region (5°S–5°N, 100–125°E). Here, we applied both definitions and extracted the relevant temperature data from the gridded data in PHYDA, defining both WPG definitions by subtracting the annual PHYDA-derived SST for the western Pacific boxes defined by Hoell and Funk^24^ and Cai et al.^25^ from Niño.4 SST. We used the range of uncertainties provided in the original PHYDA data^34^ to assess uncertainty ranges in our WPG reconstruction (Figs. S15 and S16). All time series were normalised relative to 1961–1990.

We validated the PHYDA based time series of the WPG, Niño3.4, and Niño.4 indices with temporal and spatial field correlations with the observed WPG from COBE2 SST^57^, Hadley Centre Sea Ice and Sea Surface Temperature data set (HadISST1.0^58^), instrumental ENSO indices^2^, Walker Circulation indices for SST and SLP^9,10^, GPCC gridded rainfall station^59^ and 20th-century reanalysis rainfall data^60^.

Several palaeoclimate indices for ENSO were used to verify the PHYDA Niño3.4 and WPG-based indices beyond observational data coverage. These include the Niño3.4 index of Emile-Geay et al.^22^ and the Unified ENSO Index (UEP) from McGregor et al.^18^. In addition, Walker Circulation variability was based on indices of zonal differences in sea level pressure (SLP) and SST for grid boxes defined in Coats and Karnauskas^9^ and L’Heureux et al.^10^.

We extracted Palmer Drought Severity Indices (PDSI) from the drought atlases for Australia and New Zealand (ANZDA^38^), the North American Drought Atlas and American Southwest (NADA^39,40^), and South American Drought Atlas (SADA^42^).

Spatial correlations between physical climate data and PHYDA were computed in KNMI climate explorer^61^. Only correlations > 95% significance were considered. 31-year and 51-year running correlations were also computed in^61^ KNMI climate explorer (https://climexp.knmi.nl/) using detrended annual means with statistical significance > 95% level computed against a 1000 sample Monte Carlo simulation. The 95% confidence interval was computed for all running correlations. Extreme WPG and ENSO events were calculated based on the 90th percentile of standardized indices. We calculated 20-year means of the WPG time series in PHYDA from 1001 to 2000 and identified the three strongest positive and negative WPG multi-decadal periods. For each 20-year period we calculate the mean SST anomaly (relative to the 1001–2000 mean) to illustrate the spatial patterns of SST variability (Figs. 5, 6).

All data generated for the West Pacific Gradient and zonal sea surface temperature gradient from PHYDA will be available on the NOAA Paleoclimate data server (https://www.ncdc.noaa.gov/data-access/paleoclimatology-data).

## Supplementary Information

Supplementary Information.
