# Supplementary material for: The West Pacific Gradient tracks ENSO and zonal Pacific sea surface temperature gradient during the last Millennium
Source: Sci Rep. 2021 Oct 14;11:20395. doi: 10.1038/s41598-021-99738-3 (PMC8516908; doi:10.1038/s41598-021-99738-3)
Supplement: Supplementary file 1 — Supplementary Information. [file 41598_2021_99738_MOESM1_ESM.pdf]

Supplementary Material

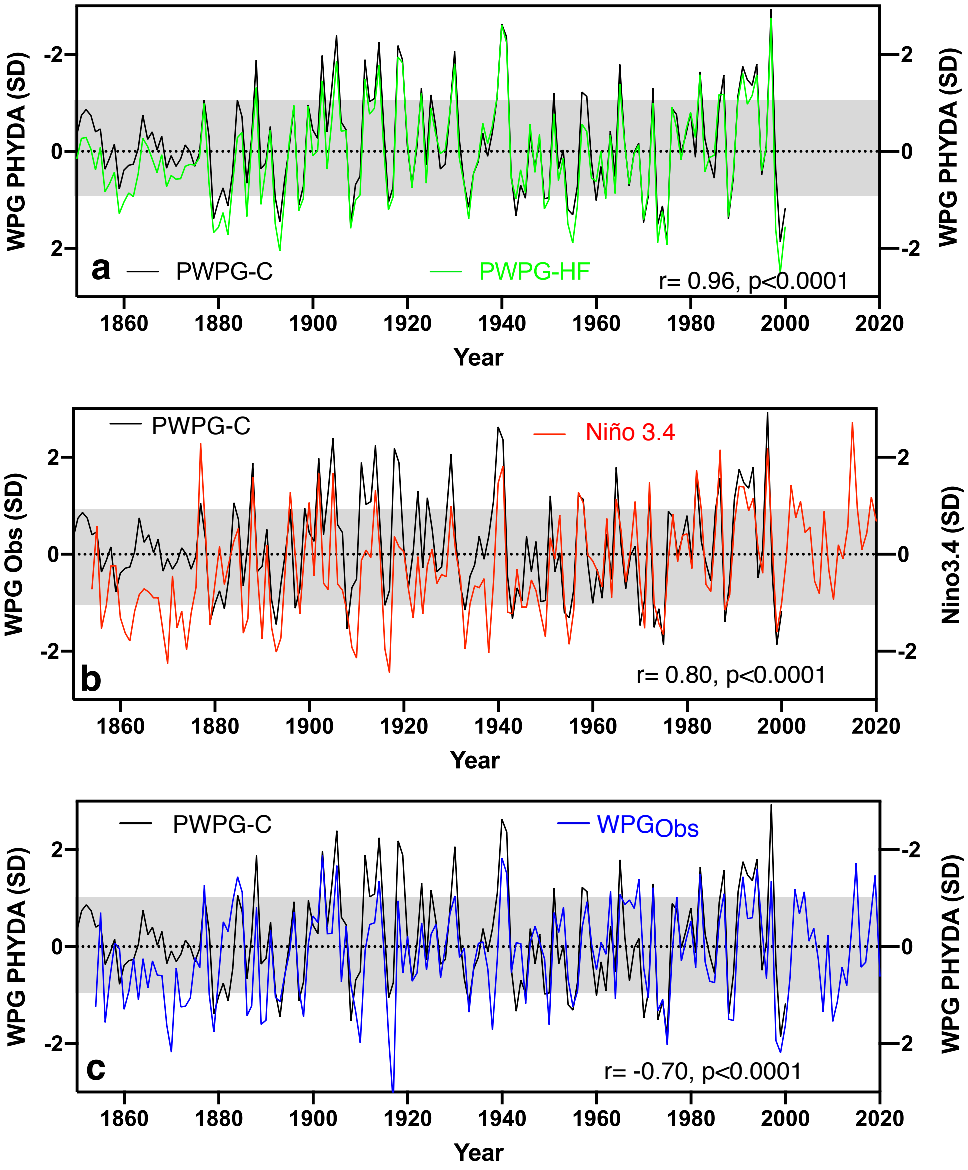

Figure S1 - PHYDA West Pacific Gradient (WPG) reconstruction following [25]Cai et al. (2015) compared to observed WPG and ENSO indices. All time series were normalised relative to 1961-1990 (SD= standard deviation). Palaeo-WPG PWPG-C based on definition by [25]Cai et al. (2015; black) compared to a) PWPG-HF ([24]Hoell & Funk, 2013; green), b) Observed Niño3.4 index ([2]Kaplan et al., 1998; red) and c) Observed West Pacific Gradient (WPG) with 1 SD (grey bar) based on [25]Cai et al. (2015; blue). Correlation coefficients (year to year) are indicated for full period of overlap after detrending.

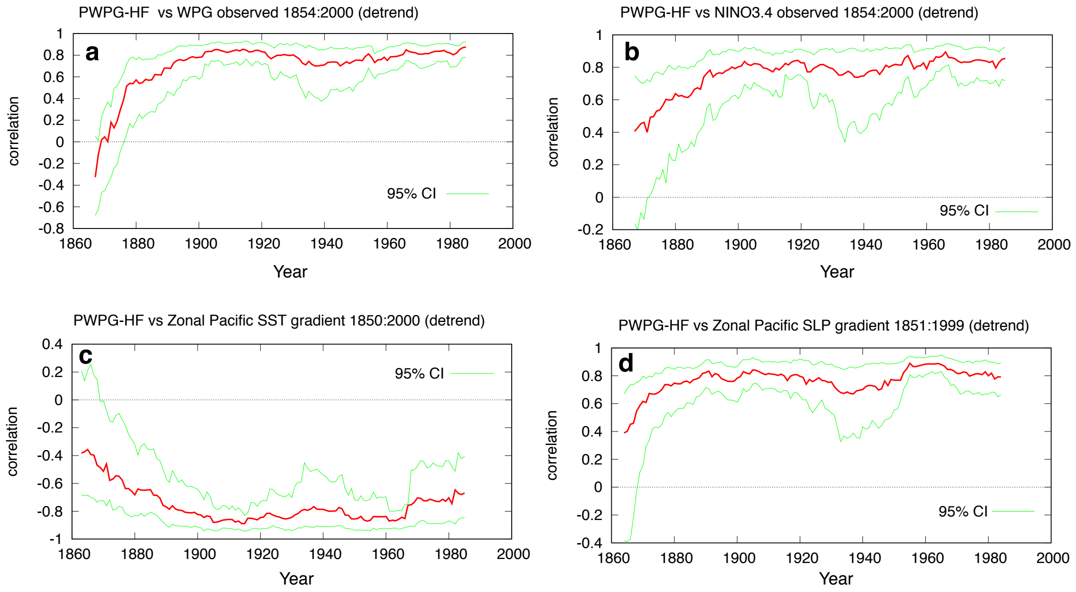

Figure S2 – PHYDA-West Pacific Gradient (PWPG-HF) reconstruction compared to observed ENSO and Walker Circulation indices. 31-year running correlations of reconstructed WPG (PWPH-HF; [24]Hoell et al., 2013) with a) observed WPG ([24]Hoell & Funk, 2013), b) Niño3.4 ([2]Kaplan et al., 1998), c) zonal SST gradient after [9]Coats & Karnauskas (2017) and d) 20th reanalysis zonal SLP gradient ([59]Slivinski et al., 2019) after after Coats & Karnauskas (2017). Correlations computed in KNMI climate explorer (detrended; [60]Trouet & Oldenborgh, 2013; https://climexp.knmi.nl/).

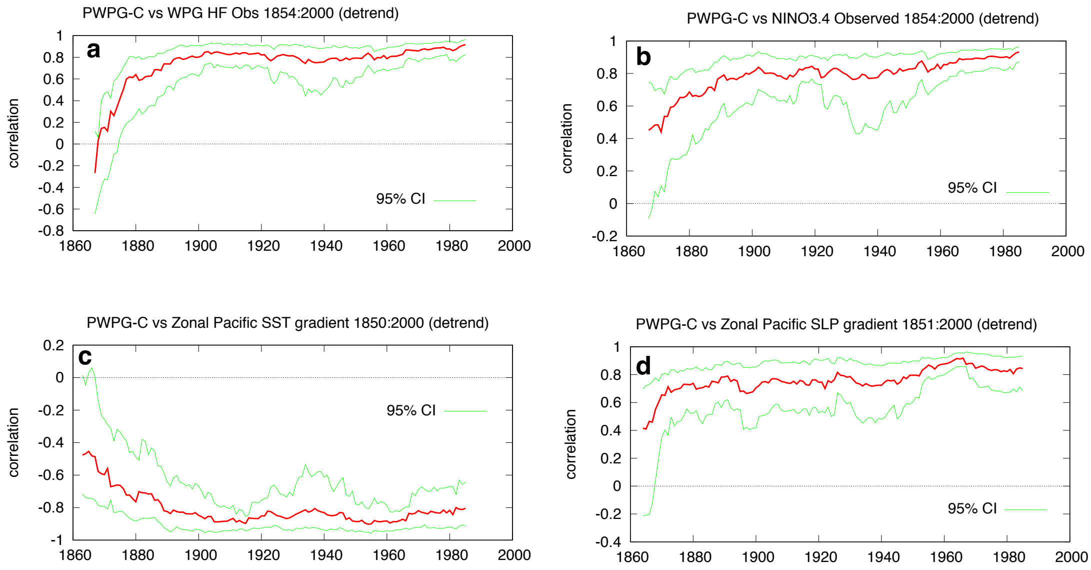

Figure S3 – PHYDA-West Pacific Gradient (PWPG-C) reconstruction compared to observed ENSO and Walker Circulation indices. 31-year running correlations of PHYDA-WPG reconstruction based on [25]Cai et al. (2015) definition (PWPG-C) with a) observed WPG ([24]Hoell & Funk, 2013), b) Niño3.4 ([2]Kaplan et al., 1998), c) zonal SST gradient after [9]Coats & Karnauskas (2017) and d) 20th reanalysis zonal SLP gradient ([59]Slivinski et al., 2019) after [9]Coats & Karnauskas (2017). Correlations computed in KNMI climate explorer (detrended; [60]Trouet & Oldenborgh, 2013; https://climexp.knmi.nl/).

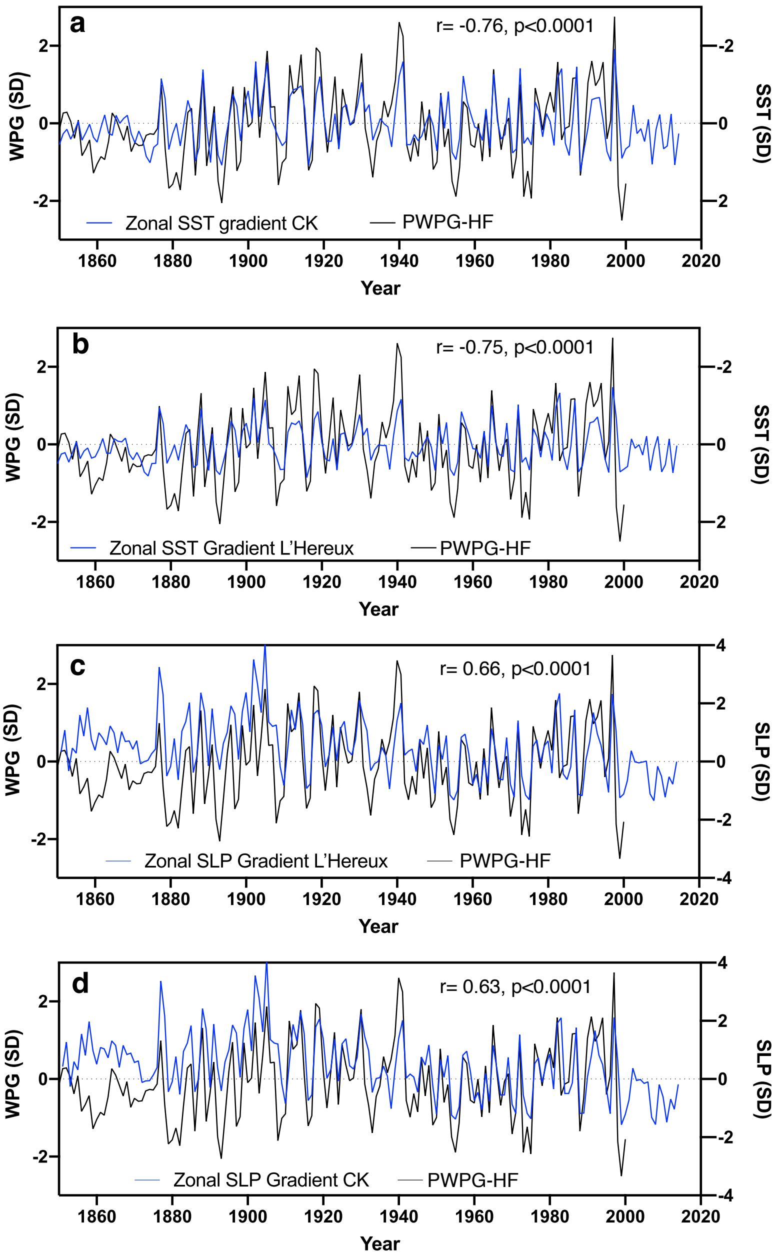

Figure S4 – Observed zonal (West – East) Pacific SST and SLP Gradient compared to West Pacific Gradient (PWPG-HF) PHYDA reconstruction. All time series were normalised relative to 1961-1990 (SD= standard deviation). PHYDA-WPG reconstruction (after [24]Hoell & Funk, 2013) compared with a) COBE 2 ([57]Hirahara et al., 2014) zonal SST gradient after [9]Coats & Karnauskas (2017), b) COBE2 zonal SST gradient after [10]L'Hereux et al. (2013), c) 20th reanalysis zonal SLP gradient after after [9]Coats & Karnauskas (2017) and d) 20th reanalysis zonal SLP gradient after [10]L'Hereux et al. (2013). Correlation coefficients (year to year) are indicated over full period of overlap for detrended data.

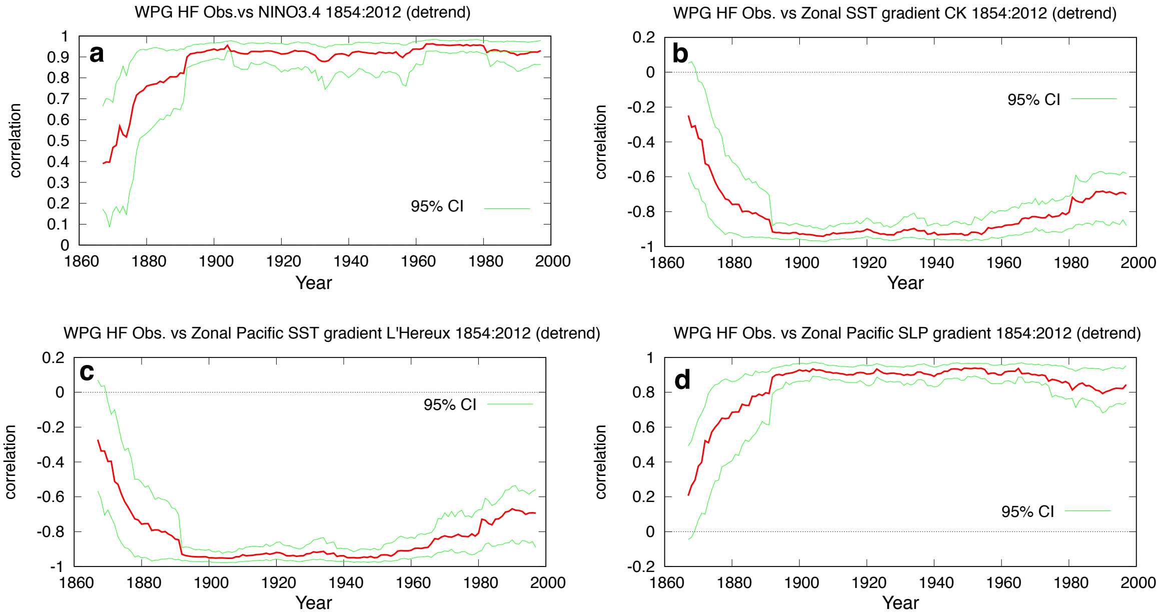

Figure S5 – Observed West Pacific Gradient (WPG) reconstruction from instrumental data compared to observed ENSO and Walker Circulation indices. 31-year running correlations of WPG from [24]Hoell & Funk (2013) compared to a) Niño3.4 ([2]Kaplan et al., 1998), b) zonal SST gradient after [9]Coats & Karnauskas (2017), c) zonal SST gradient after 1[0]L’Hereux et al. (2013) and d) 20th reanalysis zonal SLP gradient after after [9]Coats & Karnauskas (2017). Correlations computed in KNMI climate explorer (detrended; [60]Trouet & Oldenborgh, 2013; https://climexp.knmi.nl/).

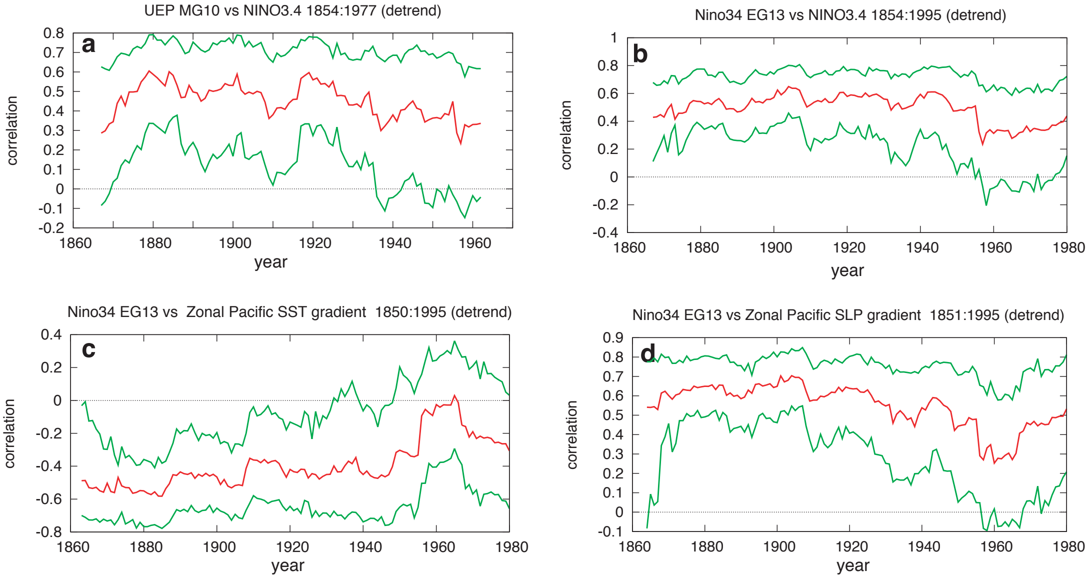

Figure S6 – Palaeo-ENSO reconstructions compared to instrumental indices of Niño3.4 ([2]Kaplan et al., 1998) and zonal SST and SLP gradients ([9]Coats and Karnauskas, 2017). a) UEP ([18]McGregor et al., 2010) with Niño3.4. [22,23]Emile-Geay et al. (2013b) Niño3.4 reconstruction correlated with b) observed Niño3.4 ([2]Kaplan et al., 1998), c) with zonal SST gradient ([9]Coats and Karnauskas, 2017) and d) with zonal SLP gradient ([9]Coats and Karnauskas, 2017). 31-year running correlations computed in KNMI climate explorer ([60]Trouet & Oldenborgh, 2013; https://climexp.knmi.nl/).

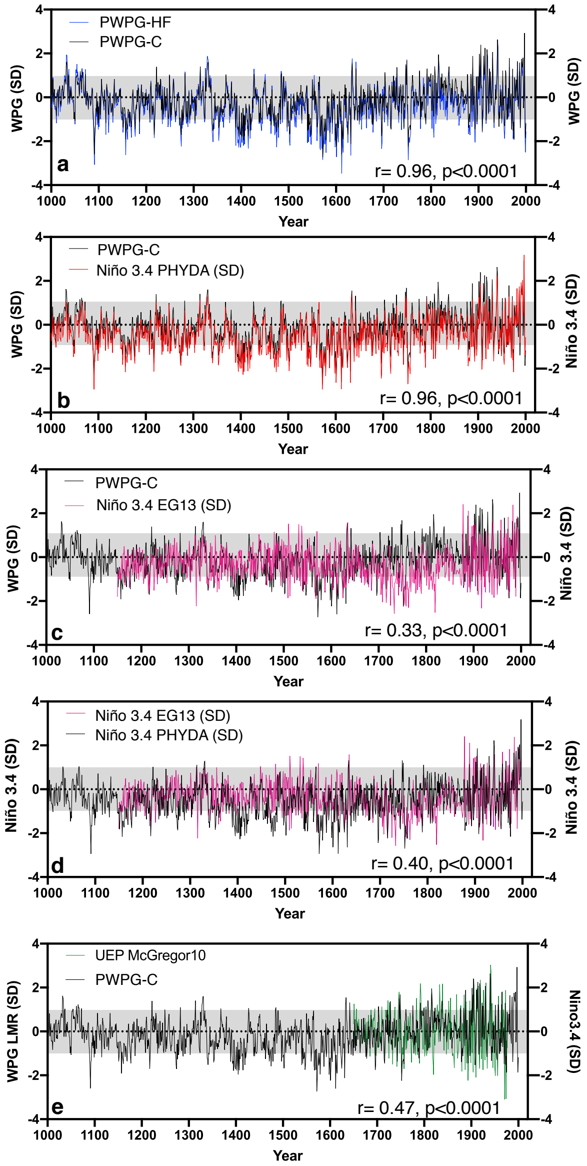

Figure S7 – Mean annual reconstruction of the WPG from the [34]PHYDA Last Millennium Reanalysis multivariate proxy network. Time series were normalised relative to 1961-1990 (SD= standard deviation). [34]PHYDA WPG based on definition of [25]Cai et al. (2015; black line; PWPG-C) with one standard deviation (SD; grey bar) compared to a) [34]PHYDA-WPG (PWPG-HF) based on definition of [24]Hoell & Funk (2013; blue), b) [34]PHYDA-based Niño3.4 index (red line), c) the Niño3.4 reconstruction (magenta line) of [22]Emile-Geay et al. (2013), d) PHYDA-based Niño3.4 index compared to the Nino3.4 reconstruction (magenta line) of [22]Emile-Geay et al. (2013), and e) PHYDA WPG based on definition of [25]Cai et al. (2015; black line) compared to the Niño3.4 reconstruction (green line) of [18]McGregor et al. (2010). Correlation coefficients (year to year) are indicated for full period of overlap after detrending.

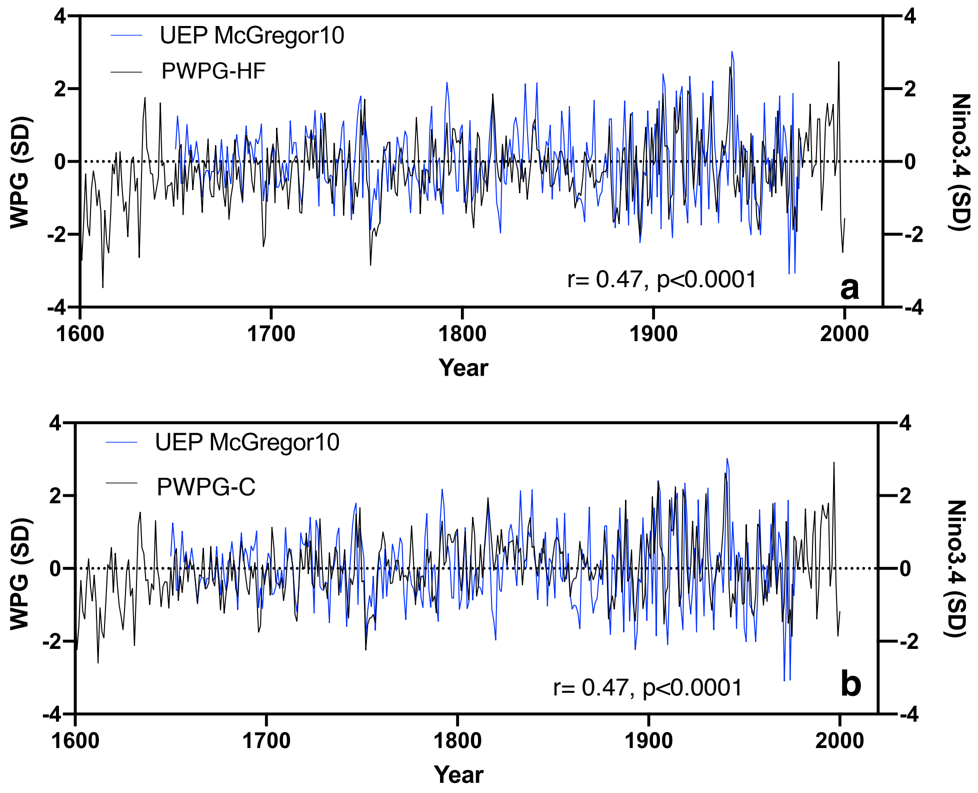

Figure S8 – Mean annual reconstruction of the WPG from [34]PHYDA based on definition of [24]Hoell et al. (2013) and Cai et al. (2015) compared to the Unified ENSO index (UEP; McGregor et al., 2010). All time series were normalised relative to 1961-1990 (SD= standard deviation). PHYDA WPG (Hoell; black line; PWPG-HF) compared to a) the Niño3.4 reconstruction from [18]McGregor et al. (2010; blue; UEP), b) PHYDA WPG ([25]Cai et al., 2015; black; PWPG-C) with the Niño3.4 reconstruction from [18]McGregor et al. (2010; blue). Correlation coefficients (year to year) are indicated over full period of overlap for detrended data.

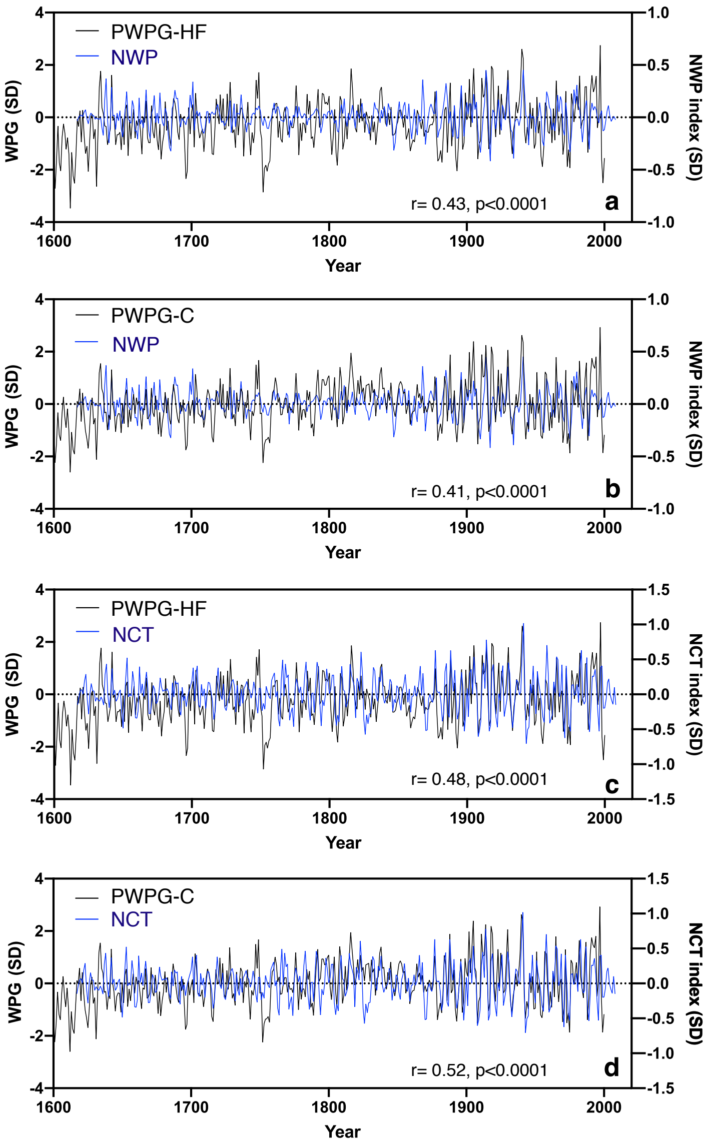

Figure S9 – Mean annual reconstruction of the WPG from the [34]PHYDA based on definition of [24]Hoell et al. (2013) and Cai et al. (2015) compared to ENSO flavor reconstructions ([47]Freund et al., 2019). All time series were normalised relative to 1961-1990 (SD= standard deviation). PHYDA WPG ([24]Hoell & Funk, 201; black line; PWPG-HF) compared to a) the Niño warm pool index (NWP) from of [47]Freund et al. (2019; blue), b) PHYDA WPG (Cai et al., 2015; black; PWPG-C) with the Niño warm pool index (NWP) from of [47]Freund et al. (2019; blue), c) PHYDA WPG ([24]black line; PWPG-HF) compared to cold tongue Niño3.4 reconstruction (NCT; blue) of [47]Freund et al. (2019), and d) PHYDA WPG (Cai; black; PWPG-C) compared to cold tongue Niño3.4 reconstruction (NCT; blue) of [47]Freund et al. (2019). Correlation coefficients (year to year) are indicated for full period of overlap after detrending.

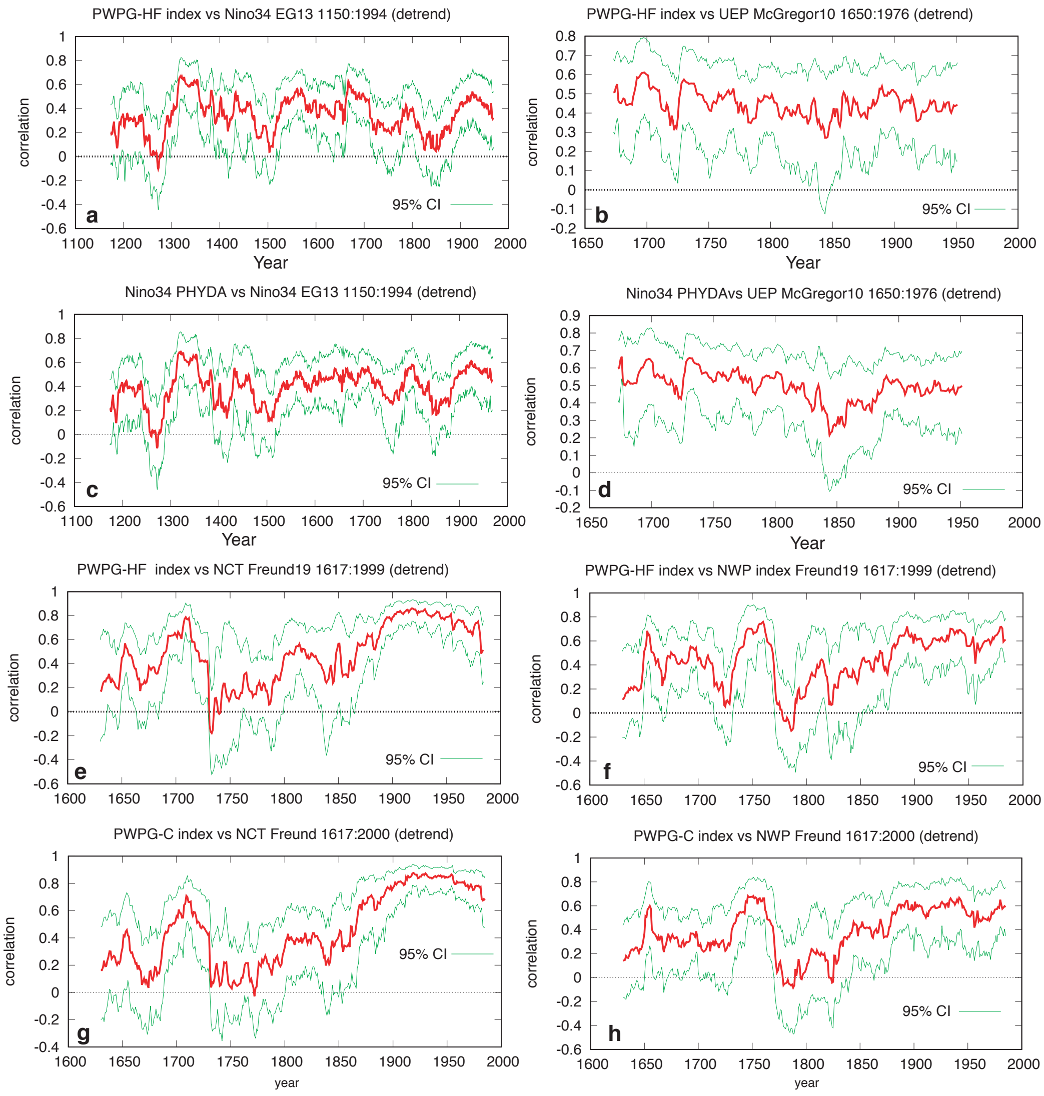

Figure S10 – [34]PHYDA-West Pacific Gradient (WPG) reconstructions compared to palaeo-ENSO reconstructions. 31-year running correlations of PHYDA-WPG reconstruction (PWPG-HF) based on [24]Hoell & Funk (2013) with a) Niño3.4 reconstruction from [25]Emile-Geay et al. (2013b: EG13), b) the Unified ENSO index (UEP) from [18]McGregor et al. (2010). 31-year running correlations of [34]PHYDA-Niño3.4 reconstruction with c) Niño3.4 reconstruction from [25]Emile-Geay et al. (2013b), d) the Unified ENSO index (UEP) from [18]McGregor et al. (2010). 31-year running correlations of PHYDA-WPG reconstruction (PWPG-HF) based on [24]Hoell & [47]Funk (2013) with d) the cold tongue ENSO (NCT) index of Freund et al. (2019) and e) the warm pool (NWP) index of [47]Freund et al. (2019). Correlations computed in KNMI climate explorer ([60]Trouet & Oldenborgh, 2013). g-h) same as e-f), but with PHYDA-WPG ([25]Cai et al., 2015) reconstruction (PWPG-C).

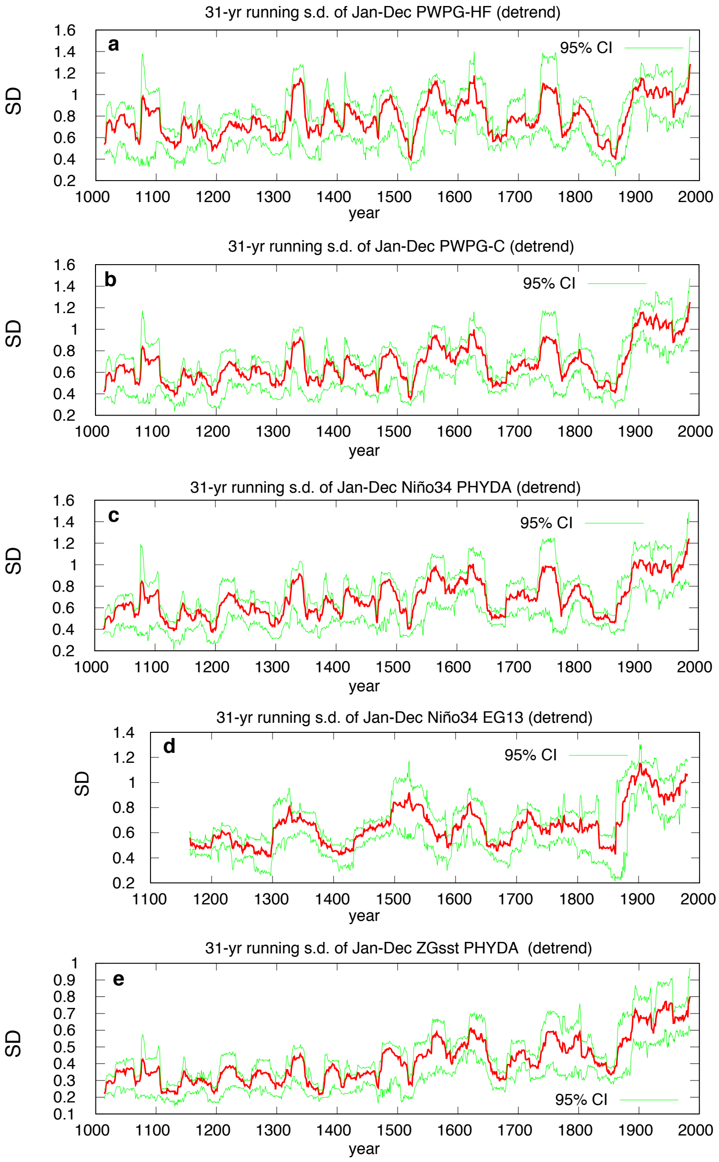

Figure S11 – 31 year running standard deviations of a) [34]PHYDA-WPG based on [24]Hoell & Funk (2013; PWPG-HF), b) [34]PHYDA -WPG based on [25]Cai et al. (2015; PWPG-C), c) [34]PHYDA based Niño3.4 index and d) Niño3.4 index of [22]Emile-Geay et al. (2013), e) PHYDA based Pacific Zonal SST gradient after Coats & Karnauskas (2017) ([34]Steiger et al., 2018). Running standard deviations and 95% confidence intervals computed in KNMI climate explorer [61](Trouet & Oldenborgh, 2013)using a Monte Carlo after detrending.

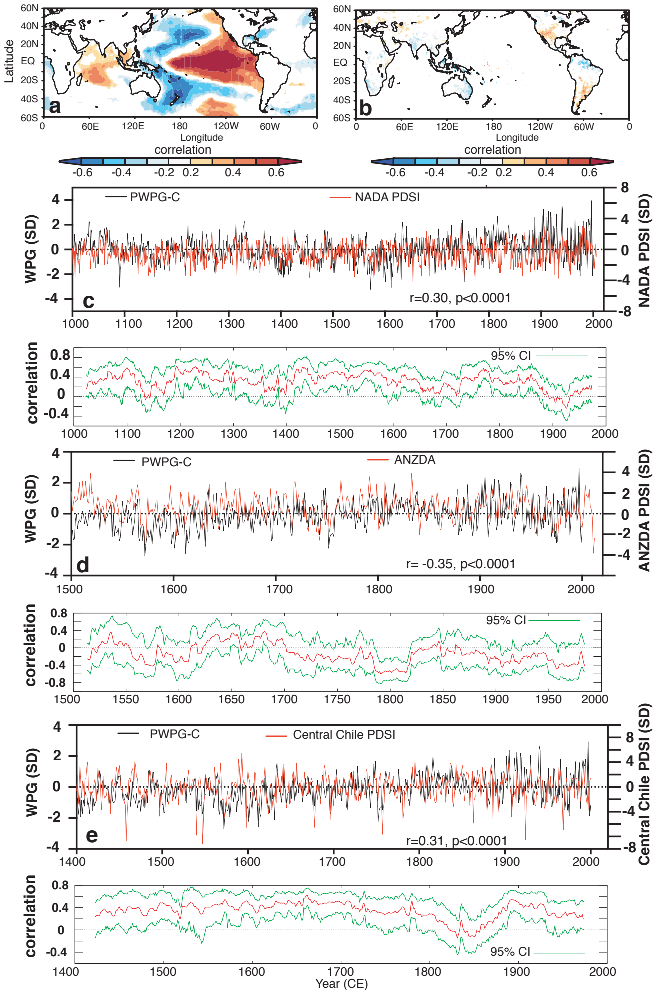

Figure S12 - Comparison between the PHYDA West Pacific Gradient (PWPG-C) (based on [25]Cai et al., 2015) reconstruction and palaeohydrological data. Time series were normalised relative to 1961-1990 (SD= standard deviation). a) Spatial correlations between the PHYDA-West Pacific Gradient (WPG) reconstruction (after [24]Hoell & Funk, 2013) and instrumental [58]HadISST (since 1870) and b) with [59]GPCC rainfall (since 1890). Only correlation >95% significance indicated in colour. Spatial correlations in a and b computed in KNMI climate explorer ([61]Trouet & Oldenborgh, 2013; https://climexp.knmi.nl/). WPG PHYDA Cai compared to c) North American drought atlas (30-40°N, 125-105°W; [39-41,61]Cook et al., 2010) and 51-year running correlation, d) Australia-Nea Zealand drought atlas (18-29°S, 140-155°E; [38]ANZDA) PDSI and 51-year running correlation, and e) Central Chile PDSI from the South America Drought Atlas (31-37°S, 70-72°W; [42]SADA) and 51-year running correlation. Correlations computed in KNMI climate explorer ([61]Trouet & Oldenborgh, 2013). Correlation coefficients (year to year) are indicated over full period of overlap for detrended data.

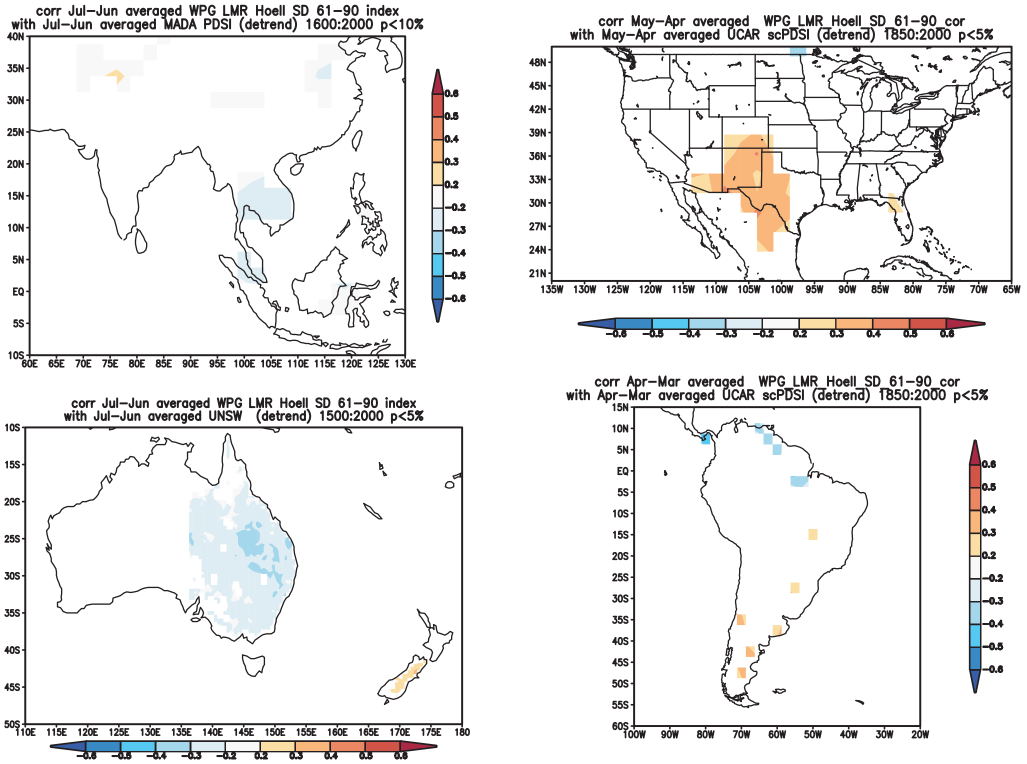

Figure S13 - Spatial correlations between PHYDA-WPG ([24]Hoell & Funk, 2013) with reconstruction of the Palmer-Drought-Severity Index (PDSI) from tree rings for a) Asia ([63]MADA), b) North America ([62]NADA), c) Australia-New Zealand ([38]ANZDA) and d) South America ([42]SADA). Correlations computed in KNMI climate explorer ([61]Trouet & Oldenborgh, 2013; https://climexp.knmi.nl/).

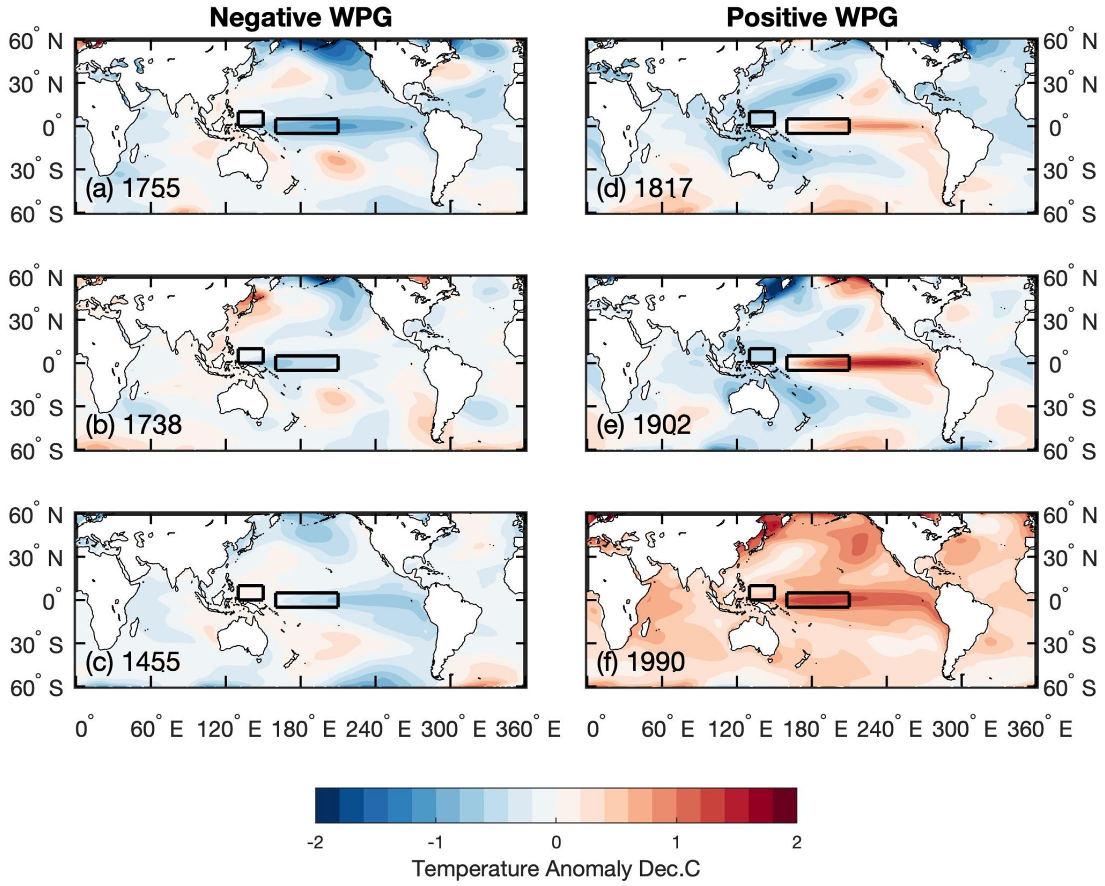

Figure S14 - Yearly mean anomalies (April to March) of the spatial surface temperature pattern in [34]PHYDA for selected time periods with left) negative and right) positive West Pacific Gradient (WPG). a) strong negative WPG associated with strong La Niña, b) strong negative WPG associated with weak La Niña and c) strong La Niña associated with weak negative WPG. d) strong positive WPG associated with weak El Niño, e) strong positive WPG associated with strong El Niño and f) weak positive WPG associated with strong El Niño. Figures created in Matlab R2020b (https://au.mathworks.com).

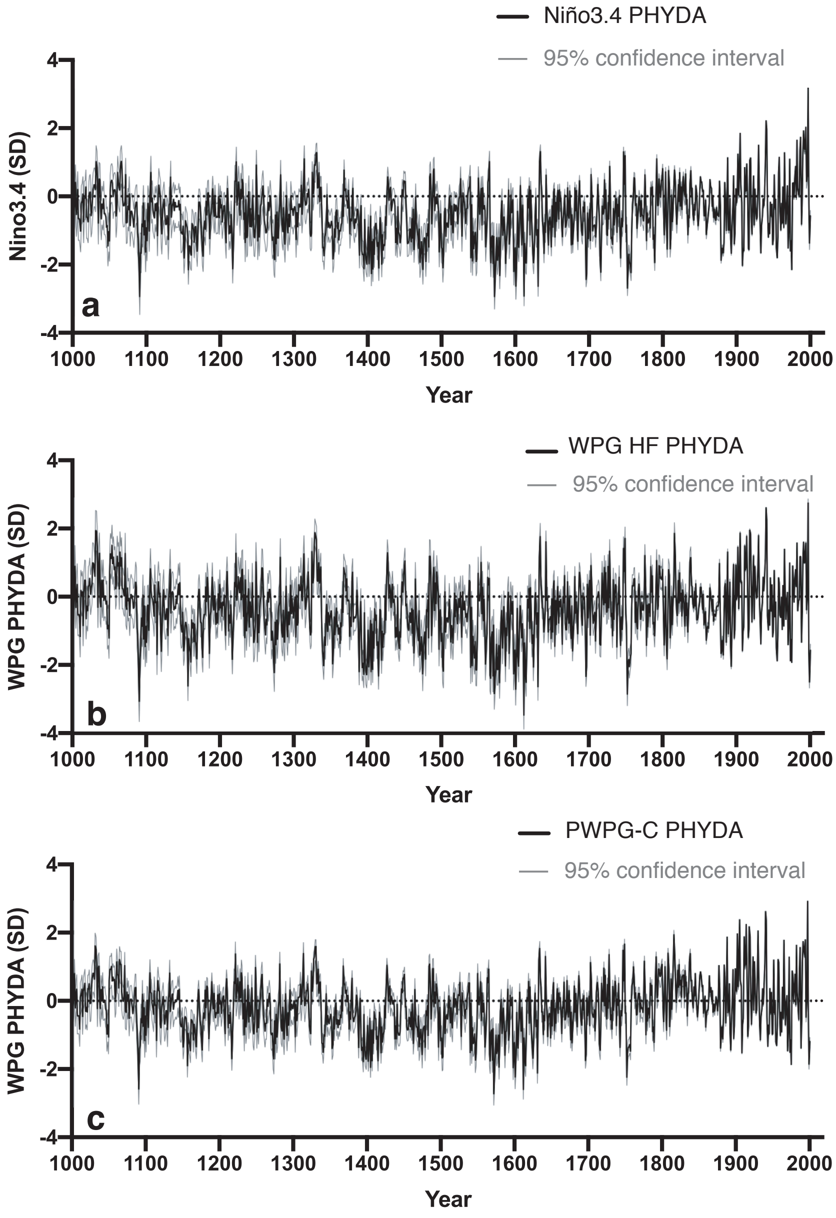

Figure S15 – [34]PHYDA Niño 3.4 and West Pacific Gradient (WPG) reconstructions (black) with 95% confidence intervals (grey) relative to 1961-1990 reference period standard deviations. a) Niño3.4, b) PWPG-HF and c) PWPG-C. All time series were normalised relative to 1961-1990 (SD= standard deviation).

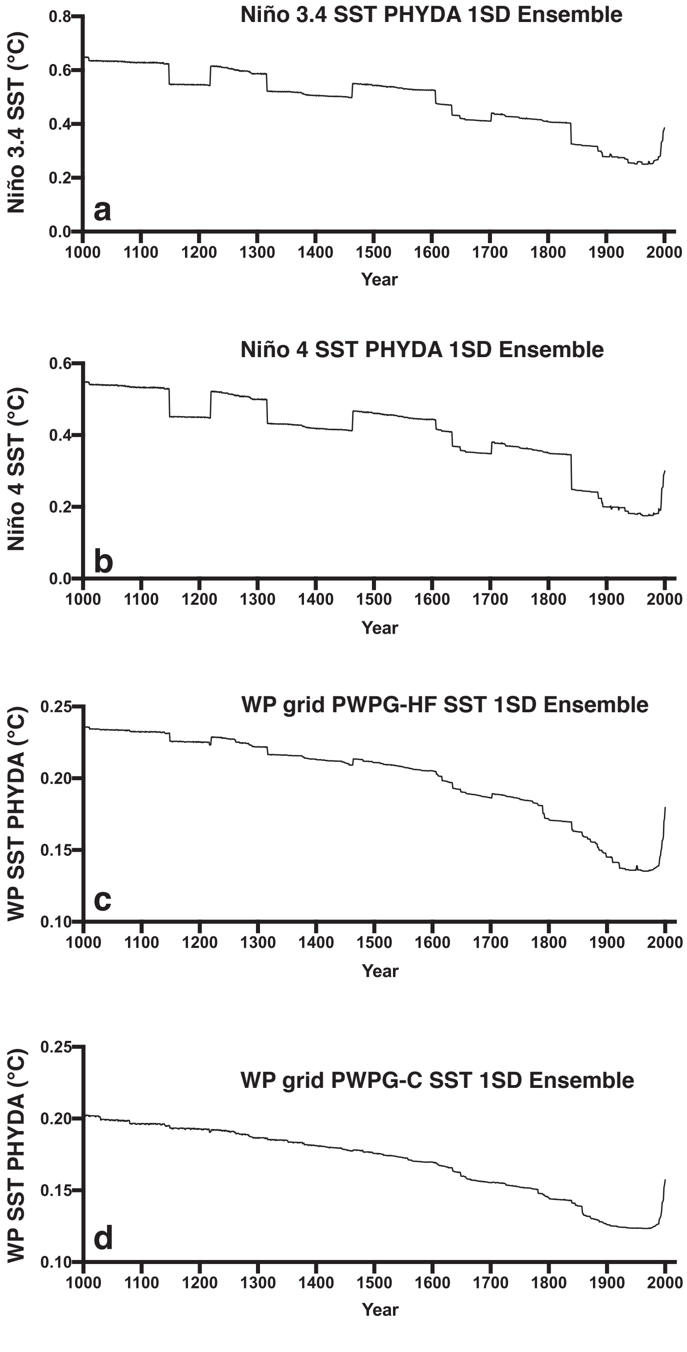

Figure S16 – Standard deviation of the ensemble reconstruction in [34]PHYDA (Steiger et al., 2018). a) Niño 3.4, b) Niño 4, c) western Pacific grid box to calculate the PWPG-HF and d) Maritime Continent grid box to calculate the PWPG-C.
